# Selective dual-mode detection of reactive oxygen species and metal ions by chemodosimetric *vs.* chelation pathways: fluorescence ‘turn-on’ with OCl^−^ and Zn^2+^/Mn^2+^, employing theoretical, practical, and bioimaging applications†

**DOI:** 10.1039/d4ra08191a

**Published:** 2025-02-28

**Authors:** Malavika S. Kumar, Avijit Kumar Das, Yatheesharadhya Bylappa, Anish Nag

**Affiliations:** a Department of Chemistry, Christ University Hosur Road Bangalore Karnataka 560029 India avijitkumar.das@christuniversity.in; b Department of Life Science, Christ University Hosur Road Bangalore Karnataka 560029 India

## Abstract

An indole-coupled diaminomaleonitrile-based fluorescent chemosensor IMA has been designed and developed for the selective detection of ROS (OCl^−^) and metal ions Zn^2+^ and Mn^2+^*via* chemodosimetric and chelation pathways respectively. The selective sensing of OCl^−^ is induced by a method of oxidatively cleaving of the imine bond of IMA, forming free indole aldehyde, which results in a 21-fold enhancement of fluorescence at 521 nm, with a detection limit of 2.8 µM. On the other hand, the selective binding of IMA with Zn^2+^ and Mn^2+^ results in chelation-induced enhanced fluorescence (CHEF) and increased intermolecular charge transfer (ICT), leading to a 4-fold and 3-fold fluorescence enhancement at 432 nm and 435 nm, with the detection limits of 12.71 µM and 17.34 µM, respectively. UV-vis spectroscopy, fluorescence, DFT study, mass spectra, ^1^H-NMR analysis, and Job's plot analysis have been used to validate the sensing mechanism of IMA with OCl^−^, Zn^2+^, and Mn^2+^. For practical applications, the binding of IMA with OCl^−^ has been utilized in the detection of commercial samples like bleaching powder and water analysis. Bio-imaging studies were conducted with IMA in the presence of OCl^−^ and Zn^2+^ using green gram seeds in a physiological medium.

## Abstract 2

An indole-coupled diaminomaleonitrile-based fluorescent chemosensor IMA has been designed and developed for the selective detection of ROS (OCl^−^) and metal ions Zn^2+^ and Mn^2+^*via* chemodosimetric and chelation pathways respectively.

## Graphical abstract

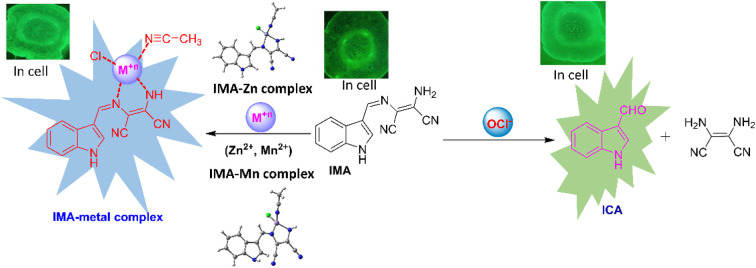

## 1. Introduction

The development of chemosensors for the detection of environmentally and physiologically active analytes has been crucial in recent years.^1^ Chemosensors offer highly selective and sensitive detection of metal ions and anions with great specificity at a very low cost. Many chemosensors have been designed based on specific host–guest interactions, such as hydrogen bonding, electrostatic forces, metal–ligand binding, hydrophobic interactions, and van der Waals forces, enabling the selective qualitative detection of various target molecules. For simplicity, affordability, and ease of use, the majority of these chemosensors have been employed in solution through spectroscopic evaluation.^2,3^ Thus, considering the fact that reactive oxygen species (ROS) are involved in many physiological and pathological processes, there is growing concern about their detection.^4–6^ Significant ROS, such as OCl^−^, have antibacterial and anti-inflammatory characteristics and play a crucial role in the human immune system.^7–9^ The fact that OCl^−^ is widely utilized in industrial domains, such as a bleaching and disinfection agent, makes its measurement all the more crucial in environmental systems.^10,11^ Numerous illnesses, including inflammatory and cardiovascular diseases, are caused by abnormally high levels of OCl^−^ in living organisms.^12–16^ Therefore, the development of practical and selective sensors to measure OCl^−^ in living systems is indispensable.^17,18^ Numerous techniques, including colorimetric, luminescent, electrochemical, and chromatographic techniques, have been effectively developed in response to this constant demand.^19^ Strong oxidizing agents like OCl^−^ have been extensively used in the development of extremely efficient fluorescent chemosensors in recent years.^20–22^ Notable reports of fluorescent probes whose spectral responses have been triggered by selective and sensitive OCl^−^-mediated oxidation involve the use of organic functionalities such as hydrazone,^23^ oxime,^24,25^ thiol,^26,27^ boronate ester,^28^ sulfonhydrazone,^29^ selenide,^30^ secondary amine, and primary amine.^31–34^

On the other hand, a substantial aspect of many biological, chemical, and environmental processes involves metal ions.^35,36^ Various metal ions, such as zinc, copper, iron, and manganese, exhibit paradoxical behavior since they can have detrimental impacts on human health whether present in excess or inadequate amounts. Manganese is one of the metal ions that is vital for many biological processes. It is necessary for the normal development of the brain and skeleton in the perinatal and neonatal stages of human life,^37^ and it functions as a cofactor in many vital enzymes, including glutamine synthetase and superoxide dismutase. However, unchecked manganese exposure to the human body can cause manganism, a disorder that causes psychosis, obsessive behaviors, depression, and mood swings.^38^ In addition to being an important nutrient, zinc is the second most prevalent transition metal ion in mammals, after iron.^39^ It is involved in numerous fundamental biological processes, including gene transcription, apoptosis, natural signal transmission or modulation, and serving as structural and catalytic cofactors. While zinc(ii) ions are generally non-toxic, excessive consumption suppresses the absorption of copper and iron, leading to deficiencies in both minerals.^40,41^ Conversely, deficiencies in zinc ions affect the kidney, liver, and brain. Since these metal ions are crucial to human health, it is necessary to address both qualitative and quantitative metal sensing.^42,43^

Thus, we have created a multifunctional chemosensor [2-(((Z)-(1*H*-indol-3-yl)methylene)amino)-3-diaminomaleonitrile] (IMA) for selective detection of anion OCl^−^ and cations Zn^2+^ and Mn^2+^ by dual pathways *via* chemodosimetric and chelation respectively. The ligand IMA has been synthesized by one step Schiff base condensation reaction between indole-3-carbaldehyde and 2,3-diaminomaleonitrile in ethanol in presence of catalytic amount of glacial acetic acid with 62% yields. The chemical structure of IMA has been confirmed by NMR and mass spectroscopic technique (Fig. S8–S9, ESI†).

## 2. Experimental

### 2.1 Materials and instrumentation

#### 2.1.1 General

Chemicals, solvents including buffer solution were purchased from Sigma-Aldrich. Melting point was determined by utilizing a hot-plate melting point equipment. On a Bruker Avance 400 MHz instrument, ^1^H-NMR spectra was recorded in DMSO-d_6_ solvent. The ^1^H–^1^H coupling constants are mentioned in Hz, and chemical shifts are specified in δ-units. UV-vis and fluorescence titration experiments were performed on UV-spectrophotometer: PerkinElmer, Lambda 30 and Shimadzu spectrofluorophotometer RF-6000 using a fluorescence cell of 10 mm path respectively. Bio-imaging experiment was measured using an epi-fluorescent microscope (LEICA DMi8 inverted microscope).

### 2.2 Synthesis and characterization of IMA

**Scheme 1:**
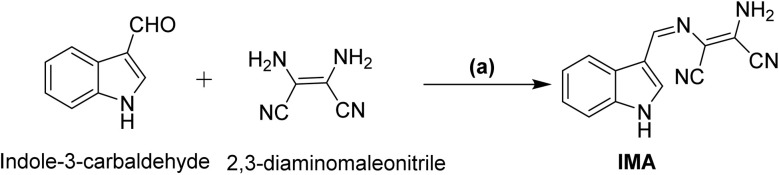
(a) Ethanol, acetic acid, r.t., 48 h.

To a solution of indole-3-carbaldehyde (300 mg, 2.06 mmol) in ethanol, 2,3-diaminomaleonitrile (222 mg, 2.05 mmol) was added followed by the addition of catalytic amount of glacial acetic acid. The resultant mixture was heated in oil bath for almost 3 min until all the reactants were dissolved and then the reaction mixture was left on stirring at room temperature for 48 h. A precipitation was separated out, filtered, washed with cold ethanol, and dried under vacuum to get a greenish yellow precipitate of IMA in pure form (Scheme 1).^44^

Yield: 300 mg, 62%. Mp: 230–235 °C. ^1^H-NMR (DMSO-d_6_, 400 MHz): 11.93 (s, 1H, –NH), 8.51 (s, 1H), 8.45 (d, 1H, *J* = 7.6 Hz), 8.15 (s, 1H), 7.46 (d, 1H, *J* = 8 Hz), 7.20 (m, 4H). Mass (*m*/*z*, %): M^+^ calculated for C_13_H_9_N_5_ is 235.086; found: 236.100 (M + H)^+^. Elemental analysis: calculated: C, 66.37; H, 3.86; N, 29.77 found: C, 66.35; H, 3.87; N, 29.78.

## 3. Results and discussion

### 3.1 Binding study of probe IMA with OCl^−^, Zn^2+^ and Mn^2+^

**Fig. 1:**
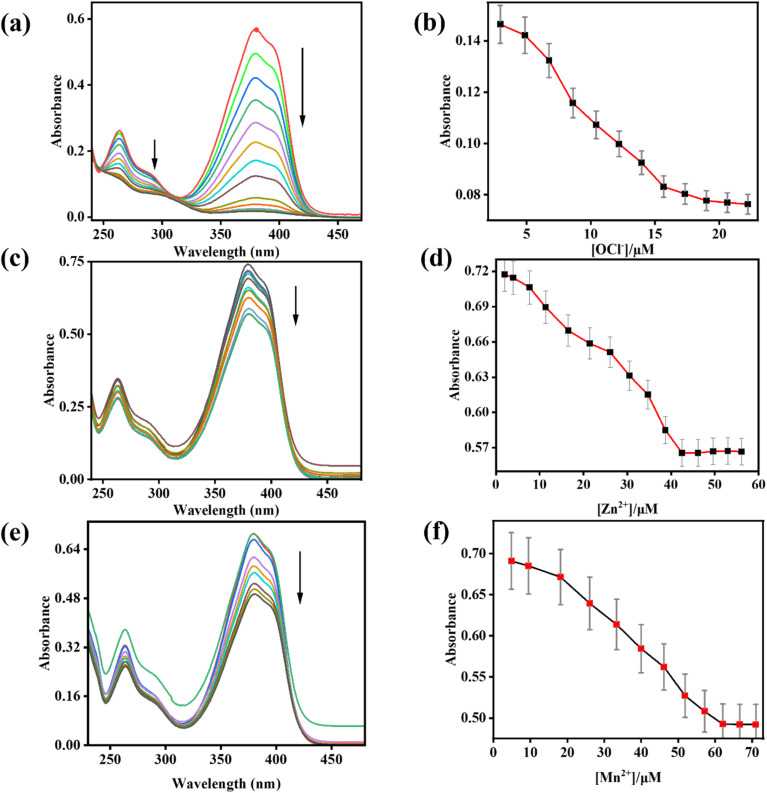
(a), (c) and (e) Absorption titration spectra of IMA (*c* = 20 µM) in presence of OCl^−^ (0–24 µM), Zn^2+^ (0–57 µM), and Mn^2+^ (0–72 µM), respectively (*c* = 200 µM). (b), (d) and (f) Variation of absorbance as a function of OCl^−^, Zn^2+^ and Mn^2+^ concentration respectively at 378 nm with error bars (error amount, 5%; *Y* error bar for both [±] deviation).

The binding study of IMA towards OCl^−^, Zn^2+^ and Mn^2+^ were performed by UV-vis and fluorescence experiments in CH_3_CN/HEPES buffer (7 : 3, v/v, pH 7.4). The ligand IMA itself exhibited two intense absorption bands at 263 nm and 378 nm respectively. However, increasing concentration of OCl^−^, the absorption bands of IMA at 263 nm and 378 nm were gradually diminished. Similarly, with incremental concentration of Zn^2+^ and Mn^2+^ to the ligand solution, the absorption signals at 263 nm and 378 nm were gradually decreased (Fig. 1).

**Fig. 2:**
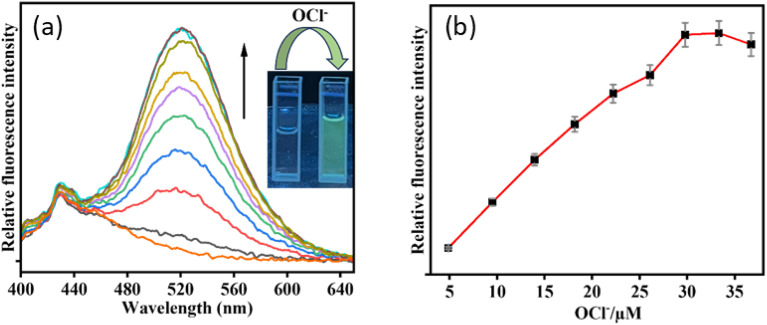
(a) Fluorescence titration experiment of IMA (*c* = 20 µM) in presence of OCl^−^ (*c* = 200 µM) (0–40 µM). Inset: changes of emission colour in absence and presence OCl^−^ under UV light irradiation. (b) Variation of fluorescence as a function of [OCl^−^] with error bars (error amount, 5%; *Y* error bar for both [±] deviation).

Fluorescence studies of IMA towards different analytes were conducted in CH_3_CN/HEPES buffer (7 : 3, v/v, pH 7.4) (*λ*_ex_ = 378 nm). Initially, IMA itself exhibited very weak fluorescence with an emission signal at 428 nm with a low quantum yield (*Φ* = 0.016). But, gradual addition of OCl^−^ to IMA solution led to 21-fold increase in fluorescence intensity with a red shifted emission peak at 521 nm (Δ*λ* = 93) ensuring a fluorescence change from colourless to greenish (*Φ* = 0.91) (Fig. 2).

**Fig. 3:**
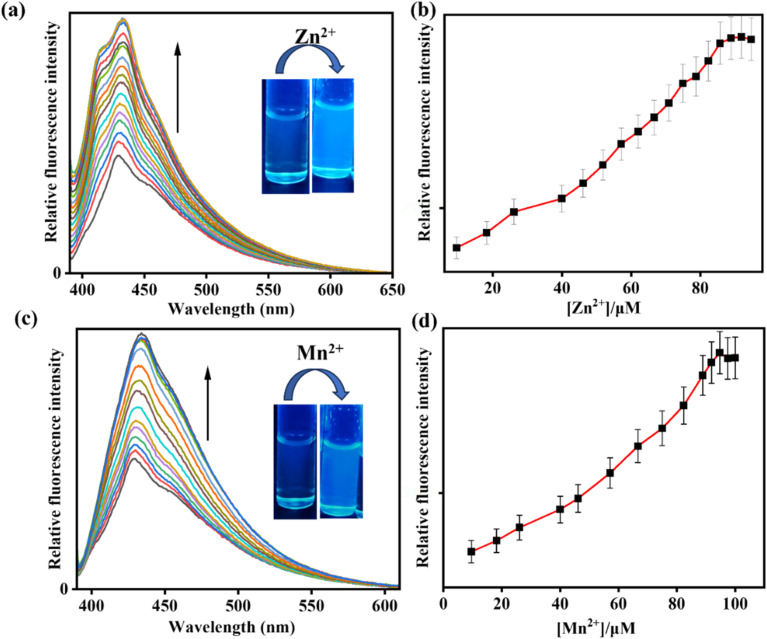
(a) and (c) Fluorescence titration experiment of IMA (*c* = 20 µM) with Zn^2+^ (0–95 µM), and Mn^2+^ (0–95 µM) respectively (*c* = 200 µM). Inset: changes of emission colours in absence and presence of Zn^2+^ and Mn^2+^ respectively under UV light irradiation. (b) and (d) Binding isotherm for variation of emission intensity with Zn^2+^ and Mn^2+^ concentration respectively (error amount, 5%; *Y* error bar for both [±] deviation).

However, the selectivity of IMA towards Zn^2+^ and Mn^2+^ was studied by the fluorescence titration in CH_3_CN/HEPES buffer (7 : 3, v/v, pH 7.4). The chemosensor IMA initially showed a weak fluorescence at 428 nm, but addition of Zn^2+^ and Mn^2+^ to receptor solution led to a bathochromic enhancement of emission signals at 432 nm (Δ*λ* = 4) and 435 nm (Δ*λ* = 7) by 4-fold and 3-fold respectively exhibiting an effective turn-on blue emission (Fig. 3). The quantum yield for IMA on binding with Zn^2+^ and Mn^2+^ is found to be 0.06 and 0.03 respectively.

The detection limits of IMA for OCl^−^, Zn^2+^, and Mn^2+^ have been determined as 2.8 µM, 12.71 µM, and 17.34 µM, respectively by using the equation DL = *K* × Sb_1_/*S*, where *K* = 3, *S* is the slope and Sb_1_ is the standard deviation of the blank solution (Fig. S2–S4, ESI†).^45^ The fast response of IMA towards OCl^−^ was determined by calculating the rate constant as 3.237 s^−1^ at 521 nm (Fig. S7†). Job's plot analysis established the 1 : 1 binding stoichiometry of IMA with Zn^2+^ and Mn^2+^ (Fig. S5 and S6, ESI†) with the notable association constants (*K*_a_) values of 3.3 × 10^2^ M^−1^ and 2.85 × 10^3^ M^−1^ respectively (Fig. S1, ESI†).^46^

### 3.2 Interference study

**Fig. 4:**
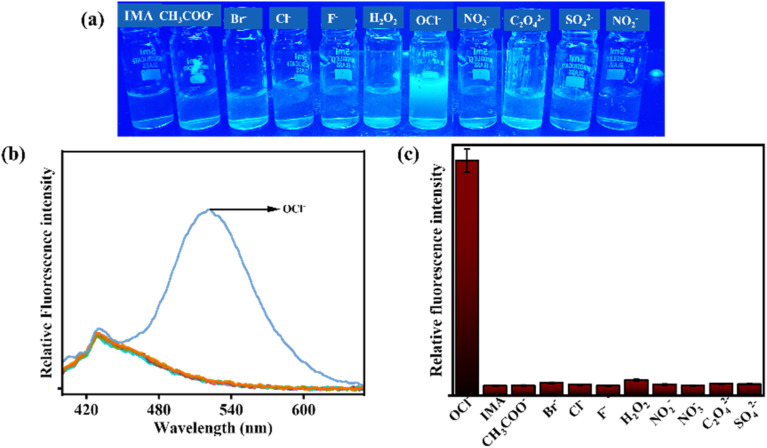
(a) Emission color changes of IMA in presence of various anions under UV light irradiation. (b) Interference fluorescence spectra of IMA (*c* = 20 µM) with various interfering anions (15 equiv.) (*λ*_ex_ = 378 nm). (c) Fluorescence responses of IMA in bar representation with different anions.

The interference studies of IMA for various interfering anions (such as Cl^−^, CH_3_COO^−^, Br^−^, F^−^, NO_2_^−^, C_2_O_4_^−^, SO_4_^2−^, H_2_O_2_, NO_3_^−^) and cations (such as Al^3+^, Cd^2+^, Fe^3+^, Fe^2+^, Hg^2+^, Mn^2+^, Cu^2+^, Ni^2+^, Pb^2+^, and Zn^2+^) were carried out in CH_3_CN : HEPES buffer (7 : 3 v/v, pH 7.4). Significantly, the selectivity of IMA towards OCl^−^ was demonstrated by the appearance of emission peak at 521 nm but no significant fluorescence changes were observed upon addition of other interfering anions, which was shown also in bar diagram (Fig. 4).

**Fig. 5:**
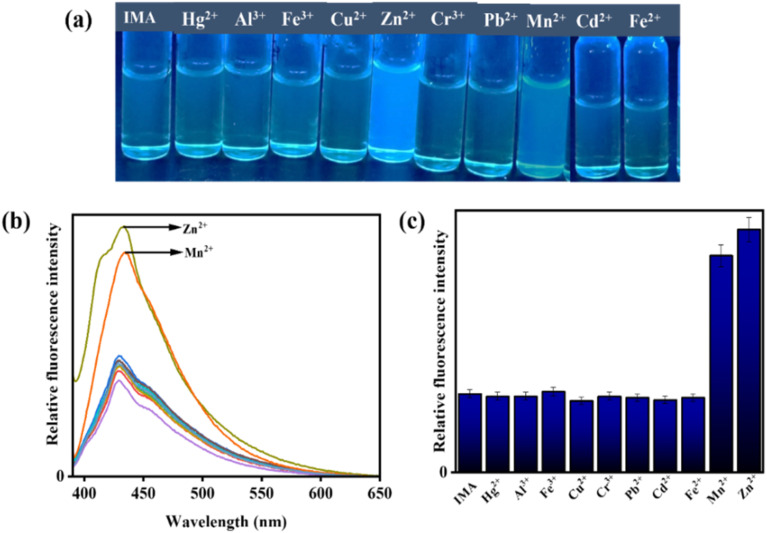
(a) Emission color changes of IMA in presence of various metal ions under UV light irradiation. (b) Interference fluorescence spectra of IMA (*c* = 20 µM) with various interfering cations (15 equiv.) (*λ*_ex_ = 378 nm). (c) Fluorescence response of IMA in bar representation with different cations.

Nevertheless, the addition of Zn^2+^ and Mn^2+^ to IMA solution resulted in a significant emission enhancement. In contrast, IMA did not exhibit any fluorescence response when other interfering metal ions were added, indicating high selectivity and sensibility of IMA for Zn^2+^ and Mn^2+^ (Fig. 5). Thus, the comparison of emission spectra (Fig. 4b and 5b) and bar diagrams (Fig. 4c and 5c) demonstrates the selectivity of IMA towards OCl^−^/Zn^2+^/Mn^2+^. In the bar diagram representations, the highest intensities, identified by maroon and blue bars, indicate the selectivity and sensitivity of IMA towards OCl^−^ and Zn^2+^ as well as Mn^2+^, respectively.

### 3.3 Binding pathway of IMA with OCl^−^, Zn^2+^ and Mn^2+^ in solution phase

**Scheme 2:**
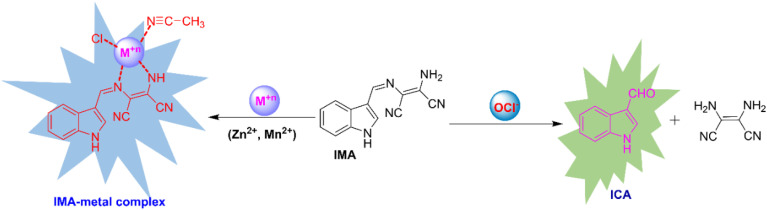
Probable pathway for sensing of OCl^−^ and M^*n*+^ (Zn^2+^, Mn^2+^) by IMA.

**Scheme 3:**
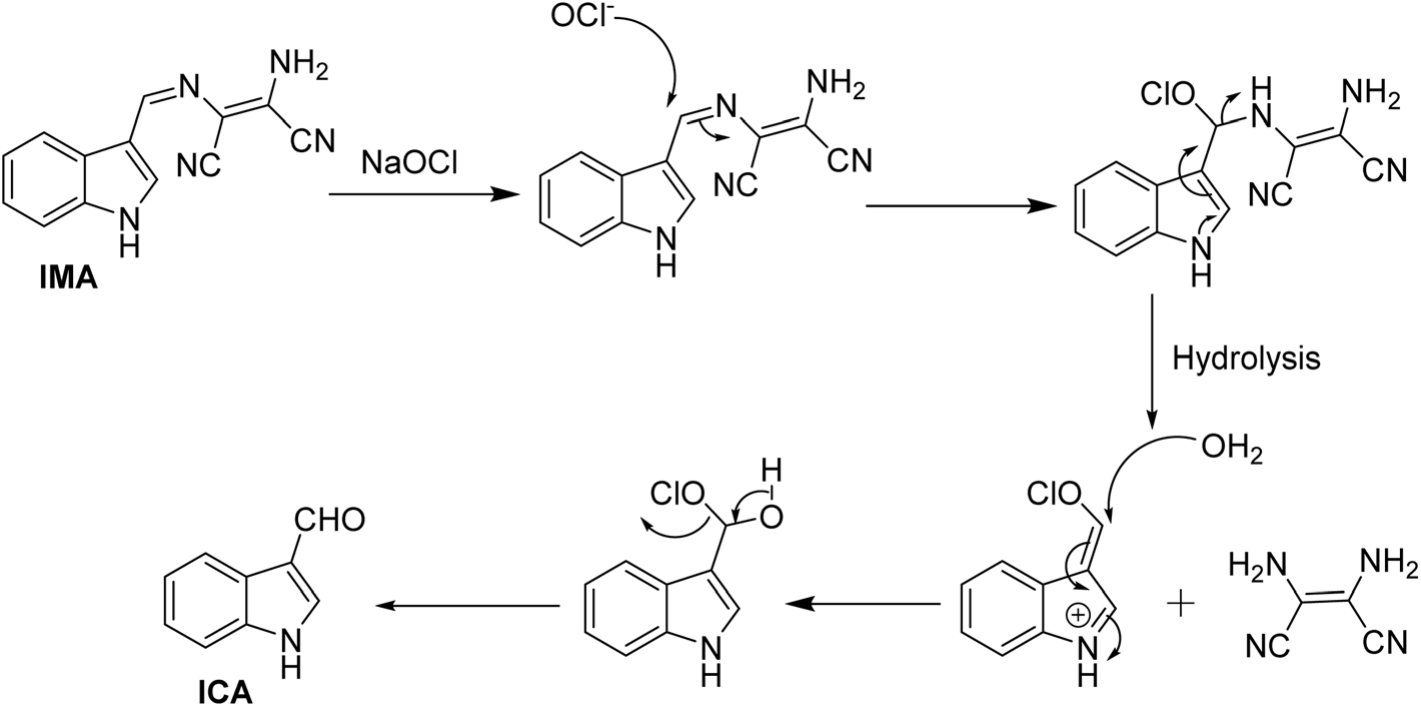
Proposed mechanism of OCl^−^ detection by IMA.

The probable binding mode of IMA with OCl^−^ and Zn^2+^, as well as Mn^2+^ in the solution phase, has been explained through the chemodosimetric and chelation pathways, respectively (Scheme 2), which result in changes in the absorption and emission wavelengths before and after the addition of the corresponding analytes (Table S2†).The initial absorbance IMA was high due to the delocalization of electrons across the molecule, which allow the molecule to absorb light in the visible or UV spectrum, leading to higher absorbance at 263 nm and 378 nm. But, the oxidative cleavage by gradual addition of OCl^−^ results the loss of conjugation reducing the molecule's ability to absorb photons at certain energies, leading to a decrease in absorbance at those wavelengths (Fig. 1a).^47^ Similarly, the binding of Zn^2+^ and Mn^2+^ with IMA leads to a decrease in absorbance due to variations in the electronic structure of the imine–metal complex causing a shift or fall in the absorbance band. This change in the electronic environment can disrupt the conjugation and lower the intensity of electronic transitions, leading to reduced absorbance of IMA (Fig. 1c and e).^47^ On contrary, IMA exhibits weak fluorescence at 428 nm due to the presence of a C=N bond that leads to non-radiative deactivation processes because the imine group introduces low-energy vibrational modes that facilitate the dissipation of energy.^48^ Moreover, the nitrogen lone pair of the imine moiety of IMA can interact with the excited state and facilitate non-radiative transitions, which results the appearance of very weak fluorescence of IMA.^49^ But, the selective detection of OCl^−^ by IMA has been demonstrated by the oxidative breakage of imine bond by OCl^−^, leading to the formation of the corresponding indole-3-aldehyde (ICA), which may subsequently lose the diaminomaleonitrile unit. Significantly, this reaction is more enhanced through the resonance conjugation by the electron donation from indole-NH, facilitating nucleophilic attack by H_2_O and resulting in the removal of 1,2-diaminomaleonitrile, thereby forming indole-3-carbaldehyde (ICA). The proposed mechanism of interaction between IMA and OCl^−^ is depicted in Scheme 3. IMA itself showed very weak fluorescence with an emission band at 428 nm with a low quantum yield. But the addition of OCl^−^ to IMA solution results fluorescence enhancement at 521 nm corresponds to the formation of indole carbaldehyde by hypochlorite mediated oxidative cleavage of imine “C=N” bond producing a greenish fluorescence (Scheme 2). Furthermore, the oxidative cleavage of imine bond of IMA and the formation of indole-3-carbaldehyde has been proved by the presence of a mass peak at *m*/*z* = 145.800 (Fig. S10, ESI†). Upon examining the ^1^H-NMR spectra of the crude product formed by the reaction of OCl^−^ with IMA, it was observed that the singlet peak corresponding to the –NH group of indole at *δ* 11.71 ppm shifted slightly downfield to *δ* 12.05 ppm due to the formation of free indole-3-aldehyde. Simultaneously, a new peak appeared at *δ* 9.93 ppm, corresponding to the proton signal of the aldehyde group in the indole moiety. Simultaneously, other aromatic proton signals shifted to downfield with higher *δ* values due to OCl^−^ induced oxidative cleavage of IMA (Fig. S13, ESI†).

On the other hand, the co-ordination with Zn^2+^ and Mn^2+^ with imine and amine nitrogen of IMA, leads to the production of CHEF (chelation-induced enhanced fluorescence) and enhanced ICT (intermolecular charge transfer), which results the enhancement of fluorescence showing a blue emission. Additionally, the formation of the M^*n*+^–IMA complex caused a fluorescence enhancement *via* the chelation-enhanced fluorescence (CHEF) mechanism. This enhancement is attributed to increased conjugation between the indole-NH and the cyanide group of diaminomaleonitrile upon metal binding, which promotes ICT process across the π-systems, resulting in enhancement of fluorescence.^50^ To indicate donor and acceptor parts of IMA, which is responsible for ICT within the molecule, Molecular electrostatic potential (MEP) and natural bond orbital (NBO) analysis have been carried out. MEP surface is a significant technique to explain the physicochemical characteristics and molecular structure.^51^ The MEP analysis provides insight into the electrophilic (negative) and nucleophilic (positive) interactions of the IMA. It reveals critical information about the molecule's size, shape, charge density, and electronegativity. The MEP surface employs various colors to indicate regions of differing electrostatic potential: red for negative, blue for positive, and green for neutral. In the IMA molecule, the electrostatic potential ranges from −5.975 × 10^2^ a.u. to +5.975 × 10^2^ a.u., with a corresponding color gradient transitioning from red to blue.^52^ The changes in electrostatic potential on the molecular surface align with color variations, following the order red < orange < yellow < green < blue^53^ (Fig. S16†). On the other hand, NBO method offers an effective framework for examining electron density distribution and electron delocalization within the molecular orbital system of IMA.^54^ NBO analysis serves as a valuable tool for understanding inter- and intramolecular interactions by analyzing donor–acceptor orbital exchanges within the molecule IMA, based on second-order perturbation theory.^55^ The calculated donor–acceptor interactions and the key stabilization energies of IMA are presented in Table S3.† Therefore, upon complexation with Zn^2+^ and Mn^2+^ result the enhancement of CHEF and ICT exhibiting a strong ‘switch on’ of blue fluorescence (Fig. 3). The complex formation of IMA with Zn^2+^ and Mn^2+^ has been demonstrated through the appearance of a peak at *m*/*z* = 374.900 and 365.700 attributed to [Zn(IMA)(Cl)(CH_3_CN)] and [Mn(IMA)(Cl)(CH_3_CN)] respectively (Fig. S11 and S12, ESI†), which has also been verified by 1 : 1 binding stoichiometric ratio by Job's plot and DFT study.

### 3.4 Practical applications

#### 3.4.1 Sensing in commercial samples

**Fig. 6:**
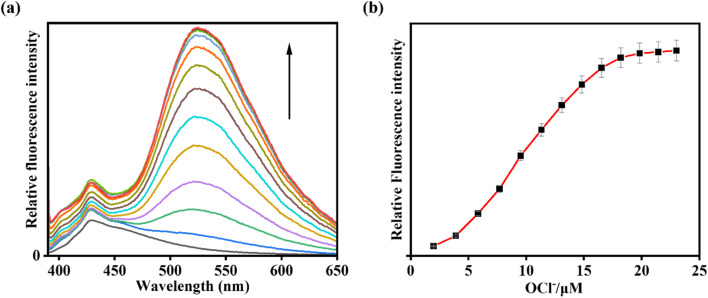
(a) Fluorescence study of IMA (*c* = 20 µM) with bleaching powder solution (*c* = 200 µM) (0–25 µM) and (b) corresponding changes of emission intensity as a function of [OCl^−^].

To employ the practical application of IMA as OCl^−^ sensor, a commercially available bleaching powder has been used to determine the presence of OCl^−^. A fluorescence experiment has been performed with IMA using bleaching powder as the source of OCl^−^, (*λ*_ex_ = 378 nm). At first, IMA showed weak fluorescence with an emission band at 428 nm. The emission intensity was enhanced by 50-fold with the addition of the bleaching powder solution to receptor solution, exhibiting a red-shifted emission peak at 521 nm (Δ*λ* = 93 nm) (Fig. 6) similar to what was observed during the titration of IMA with OCl^−^. The detection limit has been calculated as 1.495 µM, which makes the ligand useful to detect OCl^−^ in bleaching powder, which one of the most useful chemical household for cleaning purpose (Fig. S14†).

#### 3.4.2 Water analysis

**Fig. 7:**
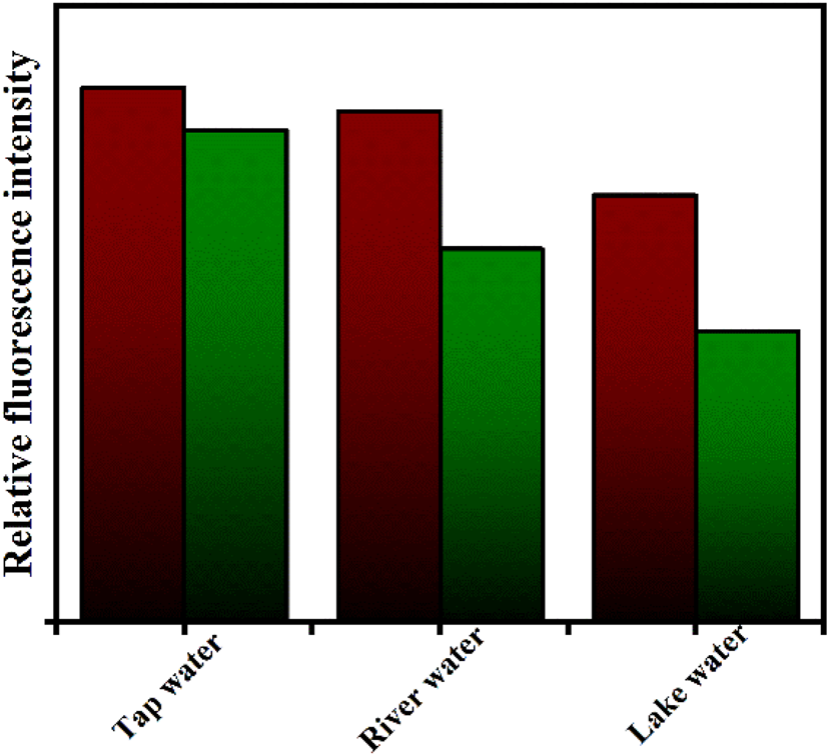
Bar graph of emission intensity of IMA in different water solution without (green bars) and with (maroon bars) OCl^−^ treatment.

Generally, OCl^−^ exist in water bodies and it may cause the damage to human and animal health. Therefore, estimating OCl^−^ in natural water sources also is very much important. Fluorescence experiment has been performed for the detection ability of IMA towards OCl^−^ and the fluorescence spectra of IMA in three different real water samples without and with the addition of incremental amount of OCl^−^ were recorded. As shown in the Fig. 7, the fluorescence enhancement noticed with addition of OCl^−^ to different water samples like lake water, river water, and tap water respectively. Detection limits of IMA in different water solutions were calculated as 5.46 µM, 3.36 µM and 2.96 µM for lake water, river water, and tap water respectively (Fig. S15†). The recovery ratios calculated using the fitting equation are presented in Table S1.† The fluorescence recovery results for OCl^−^ detection ranged from 87% to 118%, indicating that IMA is capable of monitoring micromolar concentrations of OCl^−^ in real water samples.

### 3.5 Biological study

**Fig. 8:**
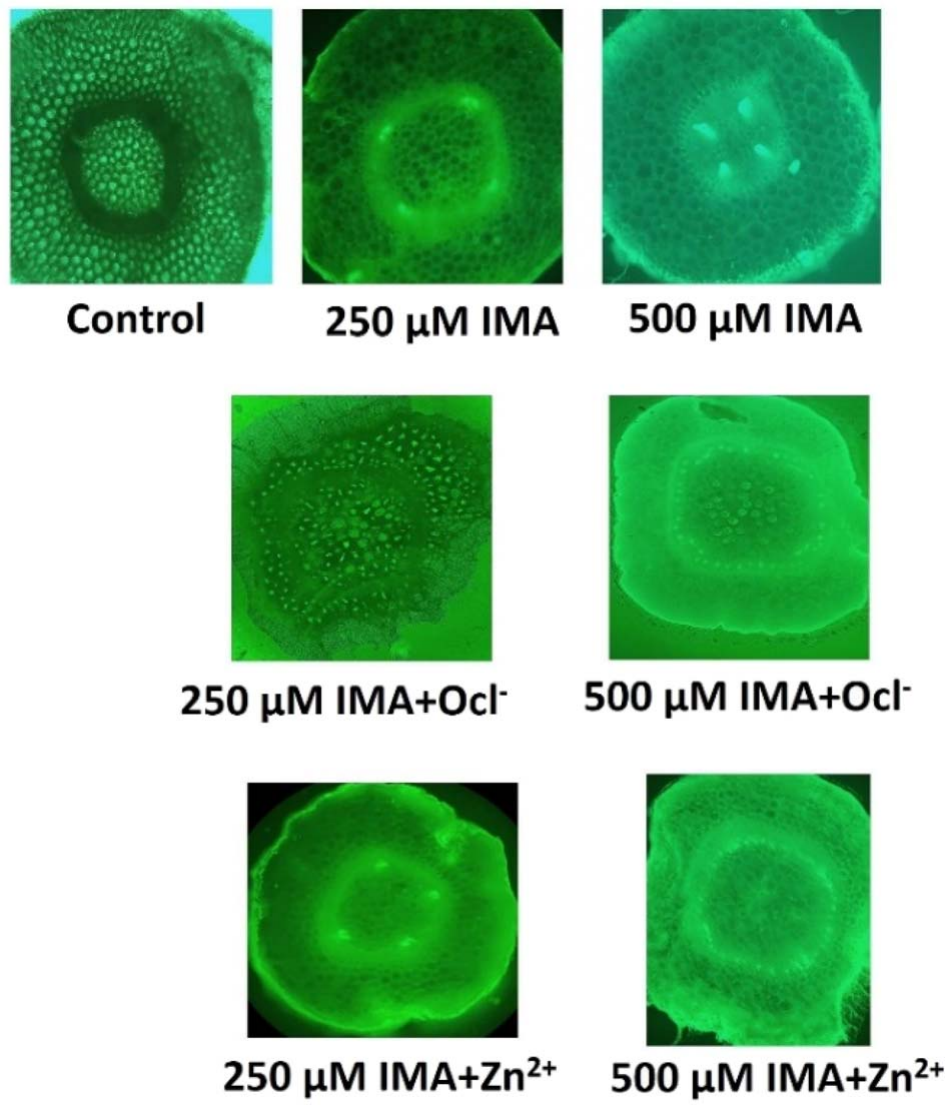
The epi-fluorescent microscopy images (490–570 nm green illumination filter) of sections of gram seed roots under low power (10×) magnification at different experimental conditions. Top panel: control (with 0.1% DMSO), 250 µM and 500 µM of IMA. Middle panel: 250 µM and 500 µM of IMA + 100 µM NaOCl, bottom panel: 250 µM and 500 µM of IMA + 100 µM Zn^2+^.

For the biological application, we have utilized the ligand IMA to detect metal ion Zn^2+^ and OCl^−^ in living plant tissue like green gram (*Vigna radiata*) seedlings. Green gram is recognized as a cost-effective, user-friendly, and readily available plant model for the preliminary assessment of OCl^−^/Zn^2+^ binding compounds. The seeds were sourced from a local market, thoroughly washed with distilled water, and prepared for sprouting. Once the root radicals grew to approximately 3 cm, the sprouted seeds (about 10 per sample) were exposed to different experimental conditions for 3 hours. After treatment, the roots were sectioned transversely, subjected to the same conditions for an additional 10 minutes, and then observed using an epi-fluorescent microscope (LEICA DMi8 inverted microscope) equipped with a 490–570 nm green illumination filter at low (10×) magnification. The transverse root sections were further analyzed under fluorescence microscopy. The results indicated that both the control and individual IMA samples showed no significant fluorescence. However, the combination of IMA and analytes (OCl^−^ and Zn^2+^) exhibited a marked increase in green fluorescence under microscopic observation (Fig. 8).

### 3.6 Theoretical study

**Fig. 9:**
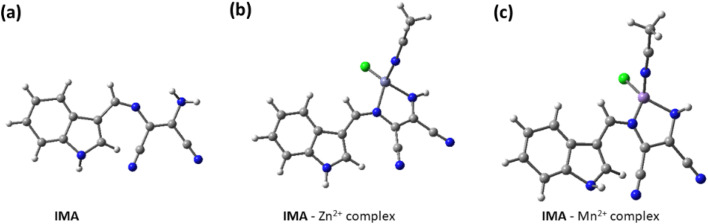
Geometry optimized molecular structures of (a) IMA and (b) IMA–Zn complex. (c) IMA–Mn complex.

To explain the binding mechanism between IMA with metal ions Zn^2+^ and Mn^2+^, we have performed the structure optimization of IMA, IMA–Zn complex and IMA–Mn complex using DFT calculations at the B3LYP level (Fig. 9). The 6-31G(d,p) basis set was used for the simple receptor (IMA), and the LANL2DZ basis set was used for the metal complex, with the calculations performed using the Gaussian 09 program. We have also measured the spatial the electron cloud distribution and the orbital energies of HOMO and LUMO for IMA, IMA–Zn complex and IMA–Mn complex. In the optimized structures of IMA–Zn complex and IMA–Mn complex, metals show square planar coordination using four co-ordinations, consisting of two nitrogens from imine and amine groups of IMA, one nitrogen of acetonitrile solvent and one chlorine as shown in the optimized structures in Fig. 9.

**Fig. 10:**
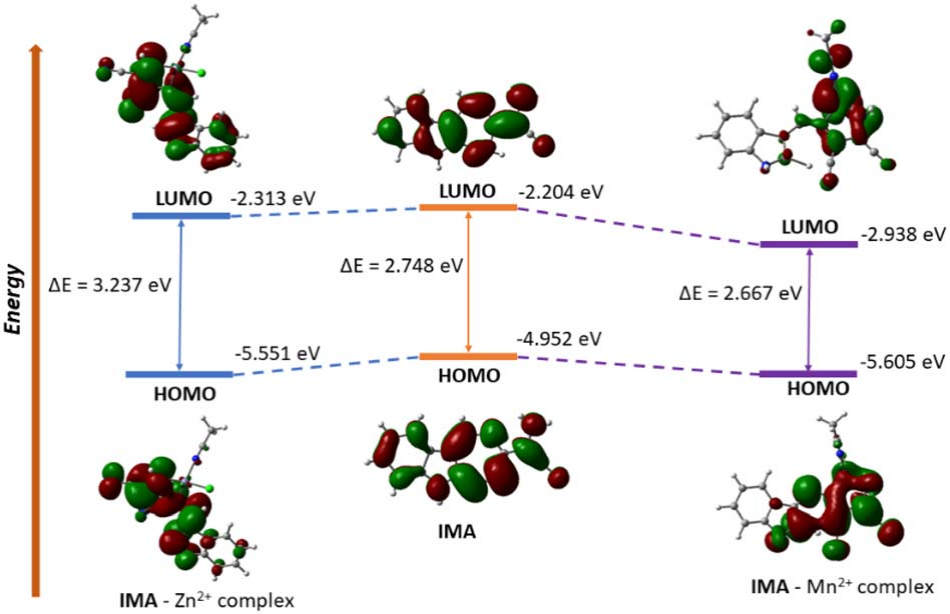
Frontier molecular orbital with energy difference of IMA, IMA–Zn complex and IMA–Mn complex.

From the optimized structure of IMA–Zn complex, the calculated distances between Zn and nitrogens of imine and amine groups of IMA are 1.41 Å, 1.94 Å respectively. The bond distances of Zn and nitrogen of acetonitrile and Zn–Cl bond distances are 2.11 Å, 2.27 Å respectively. While the bond angles between imine nitrogen, Zn with amine nitrogen and chlorine are estimated as 84.60° and 117.58° respectively and the bond angles of acetonitrile nitrogen, Zn with amine nitrogen and chlorine are 106.25° and 99.05° respectively. For IMA–Mn complex, the calculated distances between Mn and nitrogens of imine and amine groups of IMA are 1.94 Å, 1.91 Å respectively. The bond distances of Mn and nitrogen of acetonitrile and Zn–Cl bond distances are 1.86 Å, 2.26 Å respectively. While the bond angles between imine nitrogen, Mn with amine nitrogen and chlorine are estimated as 89.25° and 118.26° respectively and the bond angles of acetonitrile nitrogen, Mn with amine nitrogen and chlorine are 110.56° and 105.34° respectively. The energy gap between HOMO (−4.952 eV) and LUMO (−2.204 eV) for IMA is 2.748 eV. The frontier energy gap for IMA–Zn complex is 3.237 eV (HOMO = −5.551 eV, LUMO = −2.313 eV) and for IMA–Mn complex is 2.667 eV (HOMO = −5.605 eV, LUMO = −2.938 eV). Significantly, it is found that the energy levels of HOMO and LUMO for IMA–Zn and IMA–Mn complexes are stabilized as compared to ligand IMA itself, which is responsible for the stabilization of the complex formation (Fig. 10). The ligand IMA itself exhibits absorption maxima at 378 nm experimentally, which is good supported by theoretical absorption band at 381 nm for S0 → S2 transition (*E* = 3.2548 eV, *f* = 0.5773). The changes of the absorbance of IMA occurs on binding with Zn^2+^ and Mn^2+^ at 378 nm and from the theoretical study, the corresponding estimated absorption band has been calculated at 402 nm S0 → S3 transition (*E* = 3.0844 eV, *f* = 0.0106). Thus, the experimental results are in good agreement with the theoretical computations.

## 4. Conclusion

In conclusion, the indole-coupled diaminomaleonitrile-based fluorescent chemosensor IMA demonstrates significant potential for the selective detection of reactive oxygen species (OCl^−^) and metal ions (Zn^2+^ and Mn^2+^) through distinct sensing mechanisms. The sensor exhibits a strong fluorescence response towards OCl^−^*via* oxidative cleavage of imine bond and towards Zn^2+^ and Mn^2+^*via* CHEF and enhanced ICT pathways, with excellent sensitivity and detection limits. The sensing mechanism of IMA with OCl^−^, Zn^2+^, and Mn^2+^ has been demonstrated by UV-vis spectroscopy, fluorescence, mass spectroscopic techniques, DFT study, and Job's plot analysis. The application of various analytical techniques effectively validates these interactions, and the sensor's practical utility is demonstrated through commercial sample analysis and bio-imaging studies. These findings suggest that IMA could serve as a valuable tool in environmental and biological sensing applications.

## Data availability

The data supporting this article have been included as part of the ESI.†

## Conflicts of interest

There are no conflicts of interest to declare.

## Supplementary Material

RA-015-D4RA08191A-s001
