# Supplementary material for: Selective dual-mode detection of reactive oxygen species and metal ions by chemodosimetric vs. chelation pathways: fluorescence ‘turn-on’ with OCl− and Zn2+/Mn2+, employing theoretical, practical, and bioimaging applications
Source: RSC Adv. 2025 Feb 28;15(9):6708–17. doi: 10.1039/d4ra08191a (PMC11869208; doi:10.1039/d4ra08191a)
Supplement: RA-015-D4RA08191A-s001 [file RA-015-D4RA08191A-s001.pdf]

Supplementary Information (SI) for RSC Advances.
This journal is © The Royal Society of Chemistry 2025

Selective Dual-Mode Detection of Reactive Oxygen Species and Metal Ions by Chemodosimetric vs. Chelation Pathways: Fluorescence 'Turn-On' with OCl^- and Zn^{2+}/Mn^{2+}, Employing Theoretical, Practical, and Bioimaging Applications

Malavika S Kumar[a], Avijit Kumar Das[a]*, Yatheesharadhya bylappa[b], Anish Nag[b]

[a]Department of Chemistry, Christ University, Hosur Road, Bangalore, Karnataka, 560029 India, Email: avijitkumar.das@christuniversity.in

[b]Department of Life Science, Christ University, Hosur Road, Bangalore, Karnataka, India, 560029

CONTENTS

1. **General methods of UV-vis and fluorescence titration experiments ……………..2**
2. **Association constant determination………………………..……………………..2**
3. **Determination of fluorescence quantum yield……………..……………………........3**
4. **Calculation of the detection limit…………………………………………………3-4**
5. **Jobs plot analysis……………………………………………………………………5**
6. **Rate constant calculation…………………………………………………………5-6**
7. **^{1}H NMR and Mass spectrum of IMA……………………………………………6-7**
8. **Mass spectra of IMA with OCl^-, Zn^{2+}, Mn^{2+}…………………………………………..7**
9. **Sensing of OCl^- in commercial samples with detection limit………………………8**
10. **Detection limit calculation in water analysis………………………………………..9**
11. **Computational details…………………………………………………………10-23**
12. **References………………………………………………………………………….23**

1. General method of UV-vis and fluorescence titration:

By UV-vis method:

For UV-vis titrations, stock solution of the sensor was prepared c = 20 μM in CH_3CN-HEPES buffer (7/3, v/v, 25ºC) at pH 7.4. The solution of the guest interfering analytes like Cl^-, CH_3COO^-, Br^-, F^-, NO_2^-, $C_2O_4^{2-}$, NO_3^-, SO_4^{2-}, H_2O_2, Al^{3+}, Cd^{2+}, Fe^{3+}, Fe^{2+}, Hg^{2+}, Mn^{2+}, Cu^{2+}, Ni^{2+}, Pb^{2+}, Zn^{2+} were also prepared in the order of (c = 200 μM). Solutions of various concentrations containing sensor and increasing concentrations of cations and anions were prepared separately. The spectra of these solutions were recorded by means of UV-vis methods.

General procedure for drawing Job plot by UV–vis method:

Stock solution of same concentration of **IMA** and Zn^{2+} and **IMA** and Mn^{2+} were prepared in the order of c = 20 μM in CH_3CN-HEPES buffer (7/3, v/v, 25ºC) at pH 7.4. The absorbance in each case with different *host–guest* ratio but equal in volume was recorded. Job plots were drawn by plotting $\Delta I.X_{host}$ *vs* X_{host} (ΔI = change of intensity of the absorbance spectrum during titration and X_{host} is the mole fraction of the host in each case, respectively).

By fluorescence method:

For fluorescence titrations, stock solution of the sensor (c = 20 μM) was prepared for the titration of cations and anions in CH_3CN-HEPES buffer (7/3, v/v, 25ºC) at pH 7.4. The solution of the guest cations and anions in the order of c = 200 μM were also prepared. Solutions of various concentrations containing sensor and increasing concentrations of cations and anions were prepared separately. The spectra of these solutions were recorded by means of fluorescence methods.

2. Association constant determination:

The binding constant value of cation Zn^{2+} and $Mn^{2+}$with the sensor has been determined from the emission intensity data following the modified Benesi–Hildebrand equation, $1/\Delta I = 1/\Delta I_{max} + (1/K[C]) (1/\Delta I_{max})$. Here $\Delta I = I-I_{min}$ and $\Delta I_{max} = I_{max}-I_{min}$, where Imin, I, and Imax are the emission intensities of sensor considered in the absence of guest, at an intermediate concentration and at a concentration of complete saturation of guest where K is the binding constant and [C] is the guest concentration respectively. From the plot of $(I_{max}-I_{min})/(I-I_{min})$ against $[C]^{-1}$ for sensor, the value of K has been determined from the slope. The association constant (K_a) as determined by fluorescence titration method for sensor with Zn^{2+} and Mn^{2+} is found to be 3.3×10^2 M^{-1} and 2.8×10^3 M^{-1} (error < 10%).

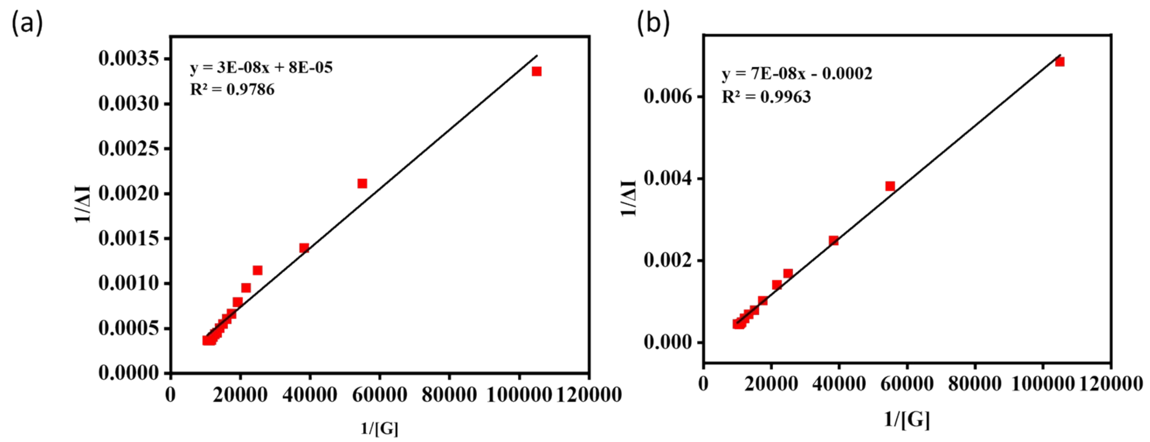

Fig. S1: Benesi–Hildebrand plot from fluorescence titration data of receptor **IMA** (c = 20 μM) with (a) Zn^{2+} and (b)Mn^{2+} including error bars (error amount, 5%; Y error bar for both [±] deviation).

3. Determination of fluorescence quantum yield:

Here, the quantum yield φ was measured by using the following equation,

$$\varphi_x = \varphi_s (F_x / F_s)(A_s / A_x)(n_x^2/ n_s^2)$$

Where,

X & S indicate the unknown and standard solution respectively, φ = quantum yield,

F = area under the emission curve, A = absorbance at the excitation wave length,

n = index of refraction of the solvent. Here φ measurements were performed using anthracene in ethanol as standard [φ = 0.27] (error ~ 10%)

4. Calculation of the detection limit:

The detection limit (DL) of **IMA** for OCl^-, Zn^{2+} and Mn^{2+} was determined from the following equation:

$DL = K^* Sb_1/S$

Where K = 2 or 3 (we take 3 in this case); Sb_1 is the standard deviation of the blank solution; S is the slope of the calibration curve.

From the graph Fig.S2, we get slope = 2.1566, and Sb_1 value is 2.0171

From the graph Fig.S3, we get slope = 28.39, and Sb_1 value is 120.359.

From the graph Fig.S4, we get slope = 24.872, and Sb_1 value is 143.819.

Thus using the formula, we get the Detection Limit for OCl^- = 2.81 μM, Zn^{2+} = 12.71μM Mn^{2+} = 17.34 μM.

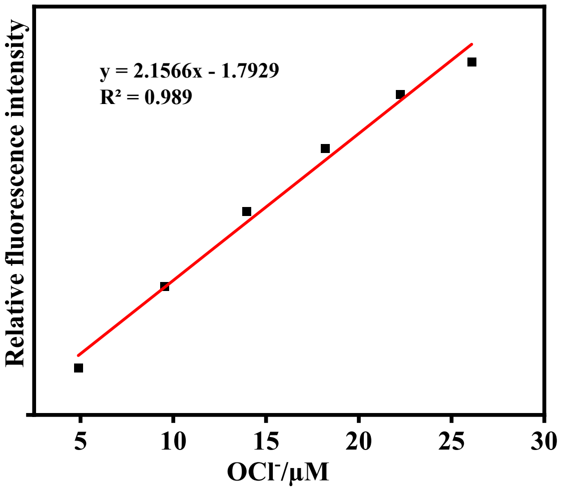

Fig. S2: Changes of fluorescence Intensity of **IMA** as a function of [OCl^-].

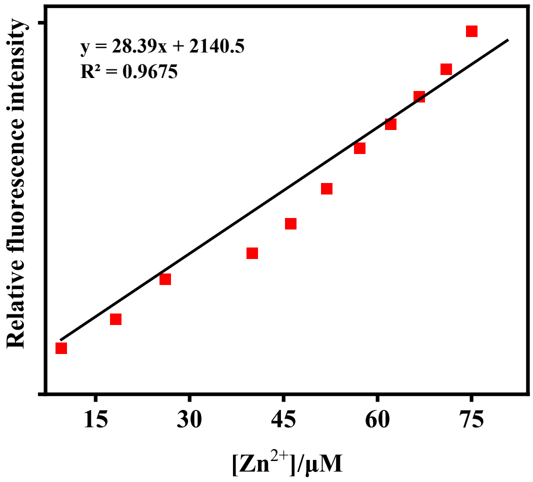

Fig. S3: Changes of fluorescence Intensity of **IMA** as a function of [Zn^{2+}].

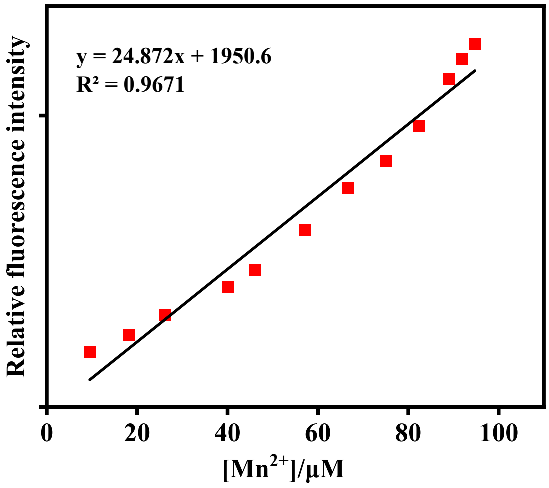

Fig. S4: Changes of fluorescence Intensity of **IMA** as a function of [Mn^{2+}].

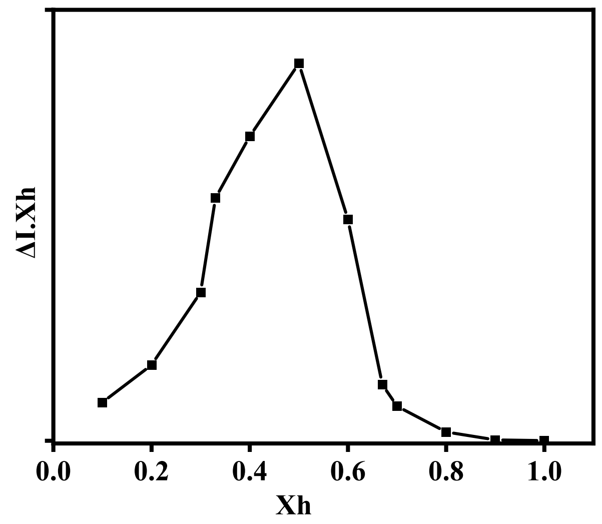

Fig. S5. Job's plot diagram of receptor **IMA** for Zn^{2+} (where Xh is the mole fraction of host and ΔI indicates the change of the intensity).

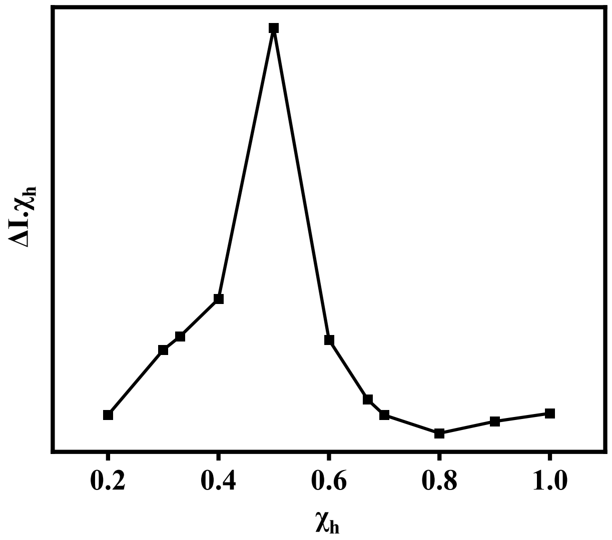

Fig. S6. Job's plot diagram of receptor **IMA** for Mn^{2+} (where Xh is the mole fraction of host and ΔI indicates the change of the intensity).

The changes of emission curve of IMA (*c* =2x10^{-5}M) at different time interval by addition of OCl^{-} (*c* =2x10^{-4}) and calculation of first order rate constant:

Fig.S7 represents the changes of emission intensity at different time interval by addition of hypochlorite. From the time vs. fluorescent intensity plot at fixed wavelength at 521 nm by using first order rate equation we get the rate constant K = slope × 2.303 = 1.4056× 2.303 = 3.237 Sec^{-1}

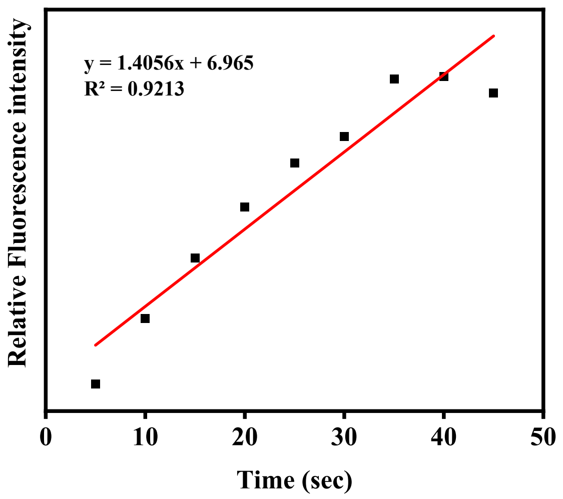

Fig. S7. The first order rate equation by using Time vs. fluorescent intensity plot at 521 nm.

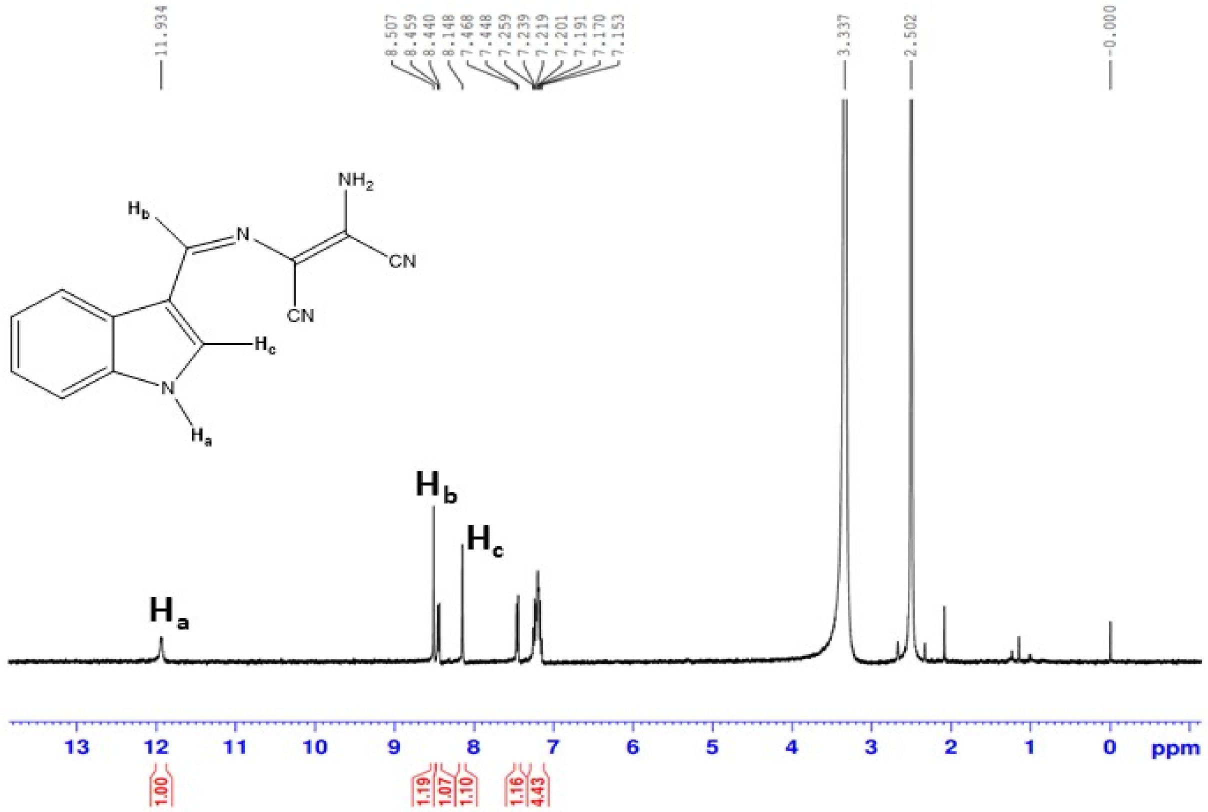

Fig.S8. ^{1}H NMR spectrum of **IMA**

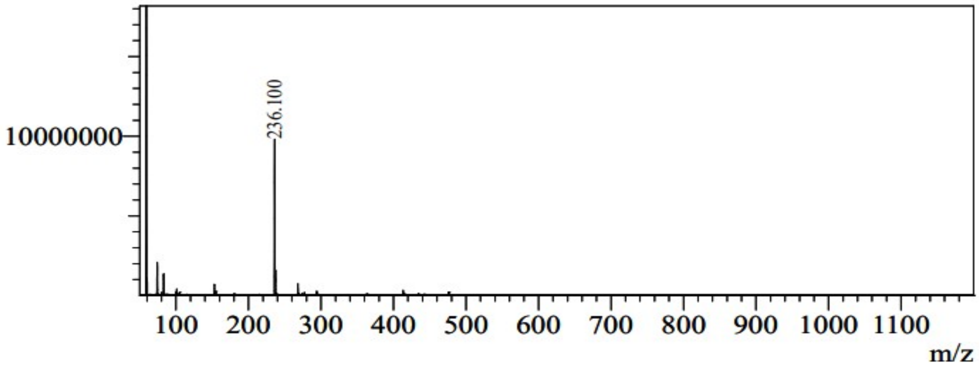

Fig.S9. Mass spectrum of **IMA**

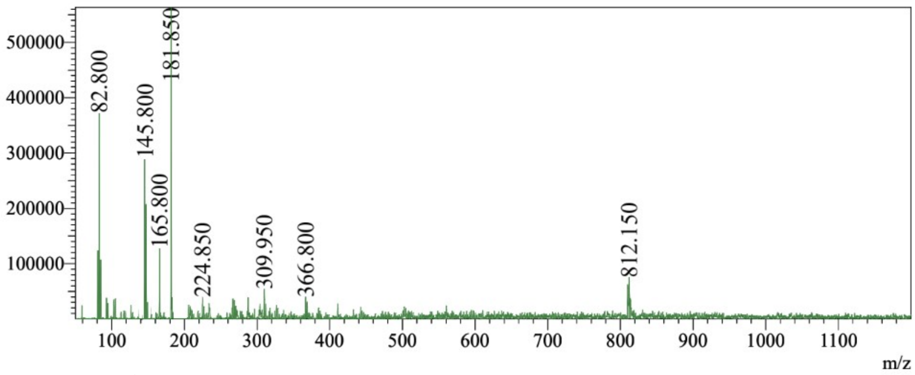

Fig. S10. Mass spectrum of **IMA** + OCl^{-}.

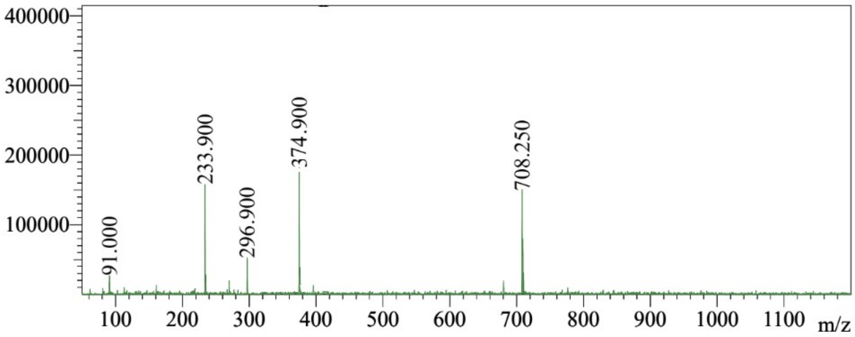

Fig.S11. Mass spectrum of **IMA** + Zn^{2+}

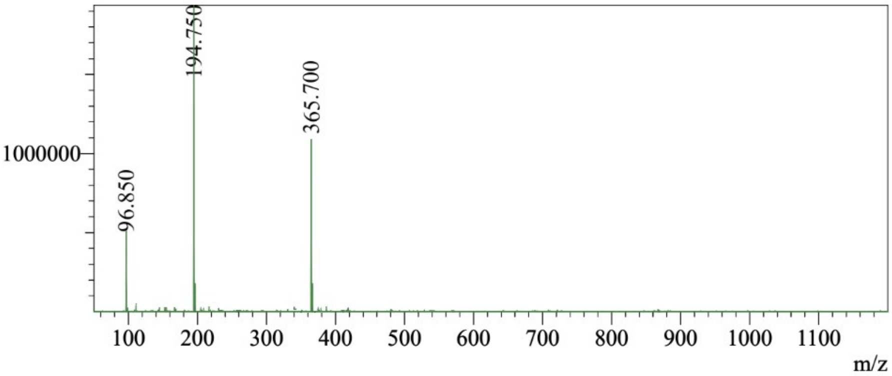

Fig.S12. Mass spectrum of **IMA** + Mn^{2+}

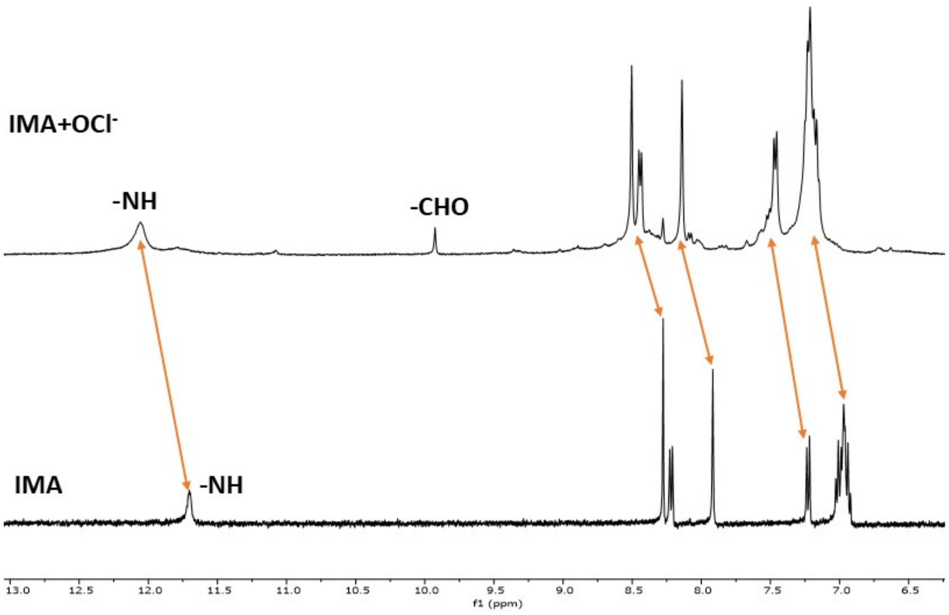

Fig.S13. ^{1}H-NMR analysis of **IMA** and **IMA**+OCl‾.

5. Sensing of OCl‾ in commercial samples and detection limit calculation

We obtained sodium hypochlorite bleach solution from a reliable supplier for our experiment, and we utilized our probe **IMA** to detect hypochlorite in it. The fluorescence spectra were recorded after the bleach solution was diluted 50 times using HEPES buffer. 10µL aliquots of this solution were gradually added to a 2×10^{-5} M solution of **IMA** in CH_3CN-HEPES buffer (1:1 v/v). Detection limit was calculated from the results.

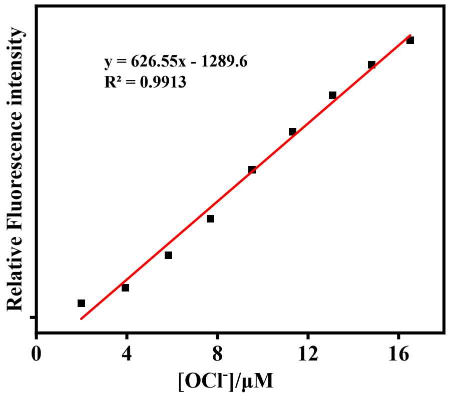

Fig. S14: Changes of fluorescence intensity of **IMA** as a function of [OCl^-].

From the graph Fig.S14, we get slope = 626.55, and Sb_1 value is 312.41.

Thus, using the formula, we get the Detection Limit for OCl^- = 1.495 µM

6. Water Analysis and detection limit calculation

The lake water and river water were collected from madiwala lake and ganga respectively in the month of January, 2023.

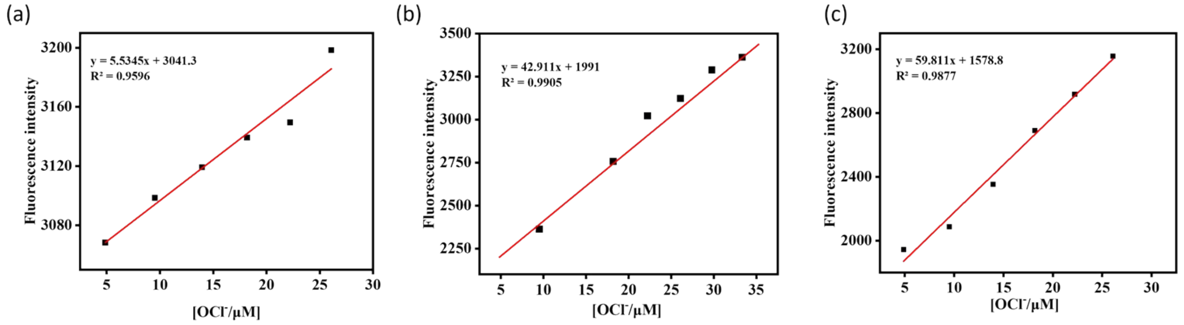

Fig. S15: Changes of fluorescence intensity of **IMA** as a function of [OCl^-] in different water samples (a) Lake water, (b) River water, (c) Tap water.

From the graphs Fig.S15, we get slopes as 5.5345, 42.911, 59.811 and Sb_1 values are 10.075, 48.122, 59.191 for (a) Lake water, (b) River water, (c) Tap water respectively.

Thus, using the corresponding formula, we get the Detection Limit for OCl^- at 5.46 µM, 3.36 µM and 2.96 µM for (a) Lake water, (b) River water, (c) Tap water respectively.

Water sample	Added OCl- (µL)	Found OCl-(µL)	Recovery (%)
Lake water	50	4.443	91.08241082
	100	10.2	107.1091043
	150	13.984	100.2437276
	200	17.863	98.25632563
River water	50	4.943	101.3325133
	100	8.5925	90.22891946
	150	16.48	118.1362007
	200	20.86	114.7414741
Tap water	50	5.095	104.4485445
	100	8.344	87.61944765
	150	12.44	89.17562724
	200	18.665	102.6677668

Table S1: Determination results of OCl^- concentration in three real water samples by **IMA**.

Analytes	Absorption wavelength of IMA before analyte addition (nm)	Absorption wavelength of IMA after analyte addition (nm)	Emission wavelength of IMA before analyte addition (nm)	Emission wavelength of IMA after analyte addition (nm)
OCl^-	378	387	428	521
Zn^{2+}	378	379	428	432
Mn^{2+}	378	380	428	435

Table S2: Photophysical properties of **IMA** in the absence/presence of analytes.

7. Computational details

Ground state electronic structure calculations in gas phase of the complexes have been carried out using DFT[1] method associated with the conductor-like polarizable continuum model (CPCM).[2] Becke's hybrid function[3] with the Lee-Yang-Parr (LYP) correlation function[4] was used for the study. The absorbance spectral properties for **IMA** and **IMA** with Zn and Mn were calculated by time-dependent density functional theory (TDDFT)[5] associated with the conductor-like polarizable continuum model and we computed the lowest 40 singlet – singlet transition. For H atoms we used 6-31+(g) basis set; for C, N, O, Zn, Mn atoms we employed LanL2DZ as basis set for all the calculations. The calculated electron-density plots for frontier molecular orbitals were prepared by using Gauss View 5.1 software. All the calculations were performed with the Gaussian 09W software package.[6] Gauss Sum 2.1 program[7] was used to calculate the molecular orbital contributions from groups or atoms. Molecular electrostatic potential (MEP) analysis is obtained from the optimised structure using B3LYP/6–31+(g) standard basis set in gas phase. Natural bond orbital (NBO) analysis of title molecule was carried out with NBO program employed in Gaussian 09 W software at B3LYP/3-21g standard level.

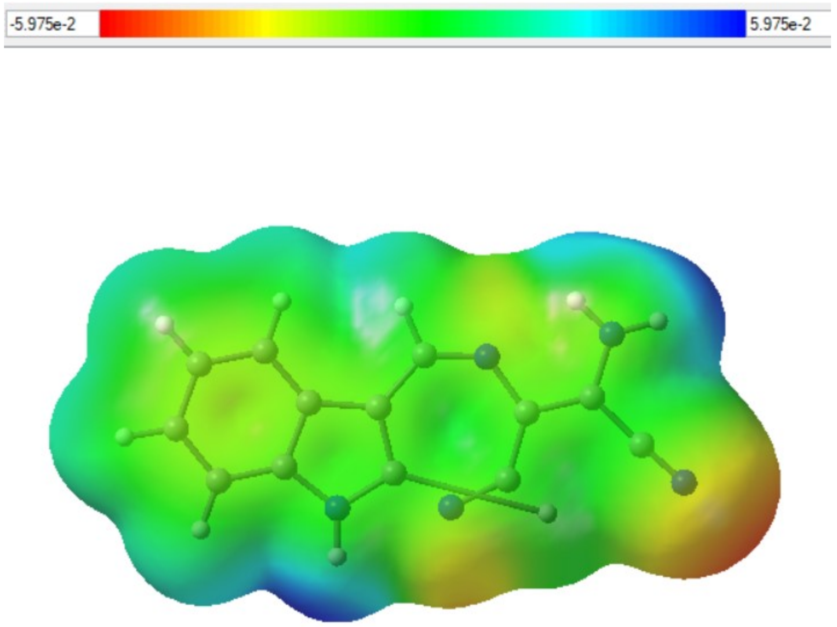

Fig. S16. Molecular electrostatic potential of **IMA**

Table S3:

Second Order Perturbation Theory Analysis of Fock Matrix in NBO Basis

NBO
File Type = .chk
Calculation Type = SP
Calculation Method = RB3LYP
Basis Set = 3-21G
Charge = 0
Spin = Singlet
Total Energy = -770.24764715 a.u.
RMS Gradient Norm = 0.00000000 a.u.
Dipole Moment = 3.1643 Debye
Threshold for printing: 0.50 kcal/mol

Donor NBO (i)	Acceptor NBO (j)	E(2)[a] kcal/mol	E(j)-E(i)[b] a.u.	F(i,j)[c] a.u.
===				
within unit 1				
1. BD (1) C 1 - C 2	/ 70. RY*(1) C 3	2.15	1.65	0.053
1. BD (1) C 1 - C 2	/ 82. RY*(1) C 6	1.43	1.94	0.047
1. BD (1) C 1 - C 2	/ 83. RY*(2) C 6	0.69	1.58	0.030
1. BD (1) C 1 - C 2	/144. BD*(1) C 1 - C 6	2.82	1.27	0.054
1. BD (1) C 1 - C 2	/146. BD*(1) C 1 - H 19	1.14	1.16	0.033
1. BD (1) C 1 - C 2	/147. BD*(1) C 2 - C 3	2.78	1.27	0.053
1. BD (1) C 1 - C 2	/149. BD*(1) C 2 - H 20	1.16	1.16	0.033
1. BD (1) C 1 - C 2	/151. BD*(1) C 3 - H 21	2.23	1.15	0.045
1. BD (1) C 1 - C 2	/157. BD*(1) C 6 - H 22	2.11	1.16	0.044

2. BD (1) C 1 - C 6	/ 66. RY*(1) C 2	0.70	1.98	0.033
2. BD (1) C 1 - C 6	/ 67. RY*(2) C 2	1.58	1.54	0.044
2. BD (1) C 1 - C 6	/ 78. RY*(1) C 5	2.62	1.94	0.064
2. BD (1) C 1 - C 6	/143. BD*(1) C 1 - C 2	2.67	1.26	0.052
2. BD (1) C 1 - C 6	/146. BD*(1) C 1 - H 19	1.27	1.18	0.035
2. BD (1) C 1 - C 6	/149. BD*(1) C 2 - H 20	1.73	1.17	0.040
2. BD (1) C 1 - C 6	/155. BD*(1) C 5 - C 6	3.49	1.27	0.059
2. BD (1) C 1 - C 6	/156. BD*(1) C 5 - C 9	4.22	1.22	0.064
2. BD (1) C 1 - C 6	/157. BD*(1) C 6 - H 22	1.39	1.17	0.036
3. BD (2) C 1 - C 6	/ 68. RY*(3) C 2	0.67	1.05	0.025
3. BD (2) C 1 - C 6	/148. BD*(2) C 2 - C 3	19.29	0.27	0.066
3. BD (2) C 1 - C 6	/153. BD*(2) C 4 - C 5	18.36	0.26	0.066
4. BD (1) C 1 - H 19	/ 66. RY*(1) C 2	0.99	1.79	0.038
4. BD (1) C 1 - H 19	/ 82. RY*(1) C 6	0.91	1.77	0.036
4. BD (1) C 1 - H 19	/143. BD*(1) C 1 - C 2	1.01	1.07	0.029
4. BD (1) C 1 - H 19	/144. BD*(1) C 1 - C 6	1.25	1.10	0.033
4. BD (1) C 1 - H 19	/147. BD*(1) C 2 - C 3	2.95	1.09	0.051
4. BD (1) C 1 - H 19	/155. BD*(1) C 5 - C 6	3.25	1.08	0.053
5. BD (1) C 2 - C 3	/ 62. RY*(1) C 1	0.79	1.98	0.035
5. BD (1) C 2 - C 3	/ 63. RY*(2) C 1	1.53	1.55	0.044
5. BD (1) C 2 - C 3	/ 74. RY*(1) C 4	1.81	1.87	0.052
5. BD (1) C 2 - C 3	/ 75. RY*(2) C 4	0.61	1.87	0.030
5. BD (1) C 2 - C 3	/143. BD*(1) C 1 - C 2	2.60	1.26	0.051
5. BD (1) C 2 - C 3	/146. BD*(1) C 1 - H 19	1.67	1.18	0.040
5. BD (1) C 2 - C 3	/149. BD*(1) C 2 - H 20	1.19	1.18	0.034
5. BD (1) C 2 - C 3	/150. BD*(1) C 3 - C 4	3.63	1.27	0.061
5. BD (1) C 2 - C 3	/151. BD*(1) C 3 - H 21	1.43	1.16	0.036
5. BD (1) C 2 - C 3	/154. BD*(1) C 4 - N 7	5.69	1.10	0.071
6. BD (2) C 2 - C 3	/ 64. RY*(3) C 1	0.67	1.05	0.025
6. BD (2) C 2 - C 3	/145. BD*(2) C 1 - C 6	17.90	0.28	0.064
6. BD (2) C 2 - C 3	/153. BD*(2) C 4 - C 5	19.55	0.27	0.068
7. BD (1) C 2 - H 20	/ 62. RY*(1) C 1	1.02	1.80	0.038
7. BD (1) C 2 - H 20	/ 71. RY*(2) C 3	0.67	1.66	0.030
7. BD (1) C 2 - H 20	/143. BD*(1) C 1 - C 2	1.04	1.08	0.030
7. BD (1) C 2 - H 20	/144. BD*(1) C 1 - C 6	2.90	1.10	0.050
7. BD (1) C 2 - H 20	/147. BD*(1) C 2 - C 3	1.19	1.10	0.032
7. BD (1) C 2 - H 20	/150. BD*(1) C 3 - C 4	3.13	1.09	0.052
8. BD (1) C 3 - C 4	/ 66. RY*(1) C 2	1.23	2.00	0.044

8. BD (1) C 3 - C 4	/ 67. RY*(2) C 2	1.30	1.57	0.041
8. BD (1) C 3 - C 4	/ 79. RY*(2) C 5	1.53	2.12	0.051
8. BD (1) C 3 - C 4	/ 86. RY*(1) N 7	0.81	2.68	0.042
8. BD (1) C 3 - C 4	/147. BD*(1) C 2 - C 3	2.82	1.30	0.054
8. BD (1) C 3 - C 4	/149. BD*(1) C 2 - H 20	1.85	1.20	0.042
8. BD (1) C 3 - C 4	/151. BD*(1) C 3 - H 21	1.38	1.18	0.036
8. BD (1) C 3 - C 4	/152. BD*(1) C 4 - C 5	5.02	1.25	0.071
8. BD (1) C 3 - C 4	/154. BD*(1) C 4 - N 7	1.62	1.13	0.038
8. BD (1) C 3 - C 4	/156. BD*(1) C 5 - C 9	1.01	1.24	0.032
8. BD (1) C 3 - C 4	/158. BD*(1) N 7 - C 8	1.08	1.14	0.031
9. BD (1) C 3 - H 21	/ 66. RY*(1) C 2	1.05	1.81	0.039
9. BD (1) C 3 - H 21	/ 75. RY*(2) C 4	1.16	1.70	0.040
9. BD (1) C 3 - H 21	/143. BD*(1) C 1 - C 2	2.78	1.09	0.049
9. BD (1) C 3 - H 21	/147. BD*(1) C 2 - C 3	1.50	1.11	0.036
9. BD (1) C 3 - H 21	/150. BD*(1) C 3 - C 4	1.30	1.10	0.034
9. BD (1) C 3 - H 21	/152. BD*(1) C 4 - C 5	3.52	1.05	0.054
10. BD (1) C 4 - C 5	/ 71. RY*(2) C 3	1.57	1.83	0.048
10. BD (1) C 4 - C 5	/ 83. RY*(2) C 6	1.55	1.57	0.044
10. BD (1) C 4 - C 5	/ 94. RY*(1) C 9	1.35	1.98	0.047
10. BD (1) C 4 - C 5	/150. BD*(1) C 3 - C 4	4.38	1.25	0.066
10. BD (1) C 4 - C 5	/151. BD*(1) C 3 - H 21	2.10	1.14	0.044
10. BD (1) C 4 - C 5	/155. BD*(1) C 5 - C 6	3.27	1.25	0.057
10. BD (1) C 4 - C 5	/156. BD*(1) C 5 - C 9	2.22	1.20	0.046
10. BD (1) C 4 - C 5	/157. BD*(1) C 6 - H 22	2.05	1.15	0.044
10. BD (1) C 4 - C 5	/159. BD*(1) N 7 - H 23	3.01	1.07	0.051
10. BD (1) C 4 - C 5	/163. BD*(1) C 9 - C 10	3.81	1.19	0.060
11. BD (2) C 4 - C 5	/ 73. RY*(4) C 3	0.87	1.04	0.030
11. BD (2) C 4 - C 5	/ 84. RY*(3) C 6	0.78	1.05	0.029
11. BD (2) C 4 - C 5	/145. BD*(2) C 1 - C 6	18.10	0.28	0.065
11. BD (2) C 4 - C 5	/148. BD*(2) C 2 - C 3	19.15	0.27	0.066
11. BD (2) C 4 - C 5	/161. BD*(2) C 8 - C 9	17.38	0.26	0.060
12. BD (1) C 4 - N 7	/ 70. RY*(1) C 3	0.59	1.77	0.029
12. BD (1) C 4 - N 7	/ 90. RY*(1) C 8	3.34	1.74	0.068
12. BD (1) C 4 - N 7	/147. BD*(1) C 2 - C 3	0.74	1.39	0.029
12. BD (1) C 4 - N 7	/150. BD*(1) C 3 - C 4	1.64	1.39	0.043
12. BD (1) C 4 - N 7	/152. BD*(1) C 4 - C 5	0.78	1.34	0.029
12. BD (1) C 4 - N 7	/155. BD*(1) C 5 - C 6	1.73	1.38	0.044
12. BD (1) C 4 - N 7	/158. BD*(1) N 7 - C 8	1.77	1.23	0.042

12. BD (1) C 4 - N 7 /159. BD*(1) N 7 - H 23 0.88 1.20 0.029

12. BD (1) C 4 - N 7 /162. BD*(1) C 8 - N 18 2.86 1.25 0.053

13. BD (1) C 5 - C 6 / 62. RY*(1) C 1 1.11 1.98 0.042

13. BD (1) C 5 - C 6 / 63. RY*(2) C 1 1.40 1.55 0.042

13. BD (1) C 5 - C 6 / 74. RY*(1) C 4 0.57 1.87 0.029

13. BD (1) C 5 - C 6 / 75. RY*(2) C 4 0.58 1.87 0.030

13. BD (1) C 5 - C 6 / 94. RY*(1) C 9 0.65 2.01 0.032

13. BD (1) C 5 - C 6 / 95. RY*(2) C 9 0.81 2.05 0.037

13. BD (1) C 5 - C 6 /144. BD*(1) C 1 - C 6 2.91 1.29 0.055

13. BD (1) C 5 - C 6 /146. BD*(1) C 1 - H 19 1.84 1.18 0.042

13. BD (1) C 5 - C 6 /152. BD*(1) C 4 - C 5 3.93 1.22 0.062

13. BD (1) C 5 - C 6 /154. BD*(1) C 4 - N 7 1.39 1.10 0.035

13. BD (1) C 5 - C 6 /156. BD*(1) C 5 - C 9 5.02 1.22 0.070

13. BD (1) C 5 - C 6 /157. BD*(1) C 6 - H 22 1.17 1.17 0.033

13. BD (1) C 5 - C 6 /160. BD*(1) C 8 - C 9 0.62 1.26 0.025

14. BD (1) C 5 - C 9 / 75. RY*(2) C 4 0.99 1.83 0.038

14. BD (1) C 5 - C 9 / 82. RY*(1) C 6 1.33 1.92 0.046

14. BD (1) C 5 - C 9 / 90. RY*(1) C 8 0.54 1.59 0.026

14. BD (1) C 5 - C 9 / 91. RY*(2) C 8 0.64 1.71 0.030

14. BD (1) C 5 - C 9 / 99. RY*(2) C 10 1.18 1.81 0.042

14. BD (1) C 5 - C 9 /144. BD*(1) C 1 - C 6 0.93 1.25 0.031

14. BD (1) C 5 - C 9 /150. BD*(1) C 3 - C 4 2.88 1.24 0.054

14. BD (1) C 5 - C 9 /152. BD*(1) C 4 - C 5 2.23 1.19 0.046

14. BD (1) C 5 - C 9 /154. BD*(1) C 4 - N 7 1.24 1.07 0.033

14. BD (1) C 5 - C 9 /155. BD*(1) C 5 - C 6 4.29 1.23 0.065

14. BD (1) C 5 - C 9 /158. BD*(1) N 7 - C 8 1.27 1.08 0.033

14. BD (1) C 5 - C 9 /160. BD*(1) C 8 - C 9 2.81 1.22 0.052

14. BD (1) C 5 - C 9 /162. BD*(1) C 8 - N 18 5.61 1.10 0.070

14. BD (1) C 5 - C 9 /163. BD*(1) C 9 - C 10 2.96 1.18 0.053

14. BD (1) C 5 - C 9 /164. BD*(1) C 10 - N 11 1.96 1.23 0.044

15. BD (1) C 6 - H 22 / 62. RY*(1) C 1 1.05 1.80 0.039

15. BD (1) C 6 - H 22 / 78. RY*(1) C 5 0.50 1.77 0.027

15. BD (1) C 6 - H 22 / 79. RY*(2) C 5 0.85 1.92 0.036

15. BD (1) C 6 - H 22 /143. BD*(1) C 1 - C 2 3.01 1.08 0.051

15. BD (1) C 6 - H 22 /144. BD*(1) C 1 - C 6 1.33 1.11 0.034

15. BD (1) C 6 - H 22 /152. BD*(1) C 4 - C 5 3.43 1.04 0.054

15. BD (1) C 6 - H 22 /155. BD*(1) C 5 - C 6 1.37 1.09 0.035

16. BD (1) N 7 - C 8 / 74. RY*(1) C 4 3.08 1.99 0.070

16. BD (1) N 7 - C 8	/150. BD*(1) C 3 - C 4	3.39	1.40	0.062
16. BD (1) N 7 - C 8	/154. BD*(1) C 4 - N 7	1.99	1.23	0.044
16. BD (1) N 7 - C 8	/159. BD*(1) N 7 - H 23	0.98	1.22	0.031
16. BD (1) N 7 - C 8	/160. BD*(1) C 8 - C 9	1.21	1.38	0.037
16. BD (1) N 7 - C 8	/163. BD*(1) C 9 - C 10	2.63	1.34	0.053
16. BD (1) N 7 - C 8	/178. BD*(1) C 17 - N 18	1.55	1.37	0.041
17. BD (1) N 7 - H 23	/ 74. RY*(1) C 4	0.85	1.86	0.035
17. BD (1) N 7 - H 23	/ 91. RY*(2) C 8	0.95	1.74	0.036
17. BD (1) N 7 - H 23	/152. BD*(1) C 4 - C 5	1.17	1.22	0.034
17. BD (1) N 7 - H 23	/154. BD*(1) C 4 - N 7	0.74	1.10	0.026
17. BD (1) N 7 - H 23	/158. BD*(1) N 7 - C 8	0.72	1.11	0.025
17. BD (1) N 7 - H 23	/160. BD*(1) C 8 - C 9	1.42	1.25	0.038
17. BD (1) N 7 - H 23	/162. BD*(1) C 8 - N 18	0.67	1.13	0.025
18. BD (1) C 8 - C 9	/ 78. RY*(1) C 5	1.34	1.97	0.046
18. BD (1) C 8 - C 9	/ 98. RY*(1) C 10	0.90	1.45	0.032
18. BD (1) C 8 - C 9	/ 99. RY*(2) C 10	0.52	1.87	0.028
18. BD (1) C 8 - C 9	/131. RY*(2) N 18	1.77	2.37	0.058
18. BD (1) C 8 - C 9	/155. BD*(1) C 5 - C 6	3.72	1.29	0.062
18. BD (1) C 8 - C 9	/156. BD*(1) C 5 - C 9	2.89	1.24	0.054
18. BD (1) C 8 - C 9	/158. BD*(1) N 7 - C 8	0.96	1.14	0.030
18. BD (1) C 8 - C 9	/159. BD*(1) N 7 - H 23	2.60	1.11	0.048
18. BD (1) C 8 - C 9	/162. BD*(1) C 8 - N 18	1.55	1.15	0.038
18. BD (1) C 8 - C 9	/163. BD*(1) C 9 - C 10	3.29	1.24	0.057
18. BD (1) C 8 - C 9	/166. BD*(1) C 10 - H 25	0.97	1.18	0.030
19. BD (2) C 8 - C 9	/100. RY*(3) C 10	0.74	1.03	0.027
19. BD (2) C 8 - C 9	/153. BD*(2) C 4 - C 5	18.46	0.28	0.067
19. BD (2) C 8 - C 9	/161. BD*(2) C 8 - C 9	4.11	0.27	0.031
19. BD (2) C 8 - C 9	/165. BD*(2) C 10 - N 11	24.15	0.27	0.074
19. BD (2) C 8 - C 9	/179. BD*(2) C 17 - N 18	11.07	0.26	0.049
20. BD (1) C 8 - N 18	/ 90. RY*(1) C 8	0.53	1.75	0.027
20. BD (1) C 8 - N 18	/126. RY*(1) C 17	3.69	1.59	0.068
20. BD (1) C 8 - N 18	/127. RY*(2) C 17	1.26	1.97	0.045
20. BD (1) C 8 - N 18	/154. BD*(1) C 4 - N 7	1.34	1.23	0.036
20. BD (1) C 8 - N 18	/156. BD*(1) C 5 - C 9	0.79	1.34	0.029
20. BD (1) C 8 - N 18	/160. BD*(1) C 8 - C 9	2.30	1.38	0.051
20. BD (1) C 8 - N 18	/178. BD*(1) C 17 - N 18	0.98	1.37	0.033
20. BD (1) C 8 - N 18	/180. BD*(1) C 17 - H 24	1.56	1.29	0.040
21. BD (1) C 9 - C 10	/ 78. RY*(1) C 5	0.81	1.95	0.036

21. BD (1) C 9 - C 10	/ 79. RY*(2) C 5		0.71	2.10	0.035
21. BD (1) C 9 - C 10	/ 91. RY*(2) C 8		1.00	1.75	0.037
21. BD (1) C 9 - C 10	/103. RY*(2) N 11		1.50	2.41	0.054
21. BD (1) C 9 - C 10	/152. BD*(1) C 4 - C 5		0.68	1.23	0.026
21. BD (1) C 9 - C 10	/156. BD*(1) C 5 - C 9		4.21	1.22	0.064
21. BD (1) C 9 - C 10	/158. BD*(1) N 7 - C 8		1.35	1.12	0.035
21. BD (1) C 9 - C 10	/160. BD*(1) C 8 - C 9		4.08	1.26	0.064
21. BD (1) C 9 - C 10	/164. BD*(1) C 10 - N 11		1.50	1.27	0.039
21. BD (1) C 9 - C 10	/166. BD*(1) C 10 - H 25		0.80	1.16	0.027
22. BD (1) C 10 - N 11	/ 94. RY*(1) C 9		1.21	2.20	0.046
22. BD (1) C 10 - N 11	/ 98. RY*(1) C 10		0.89	1.62	0.034
22. BD (1) C 10 - N 11	/106. RY*(1) C 12		2.93	2.05	0.069
22. BD (1) C 10 - N 11	/110. RY*(1) C 13		0.55	2.05	0.030
22. BD (1) C 10 - N 11	/156. BD*(1) C 5 - C 9		1.48	1.41	0.041
22. BD (1) C 10 - N 11	/163. BD*(1) C 9 - C 10		1.59	1.40	0.043
22. BD (1) C 10 - N 11	/166. BD*(1) C 10 - H 25		0.57	1.35	0.025
22. BD (1) C 10 - N 11	/167. BD*(1) N 11 - C 12		1.36	1.29	0.038
22. BD (1) C 10 - N 11	/168. BD*(1) C 12 - C 13		0.88	1.45	0.032
23. BD (2) C 10 - N 11	/161. BD*(2) C 8 - C 9		8.43	0.32	0.051
23. BD (2) C 10 - N 11	/169. BD*(2) C 12 - C 13		14.20	0.32	0.065
24. BD (1) C 10 - H 25	/ 95. RY*(2) C 9		0.94	1.88	0.038
24. BD (1) C 10 - H 25	/102. RY*(1) N 11		1.07	1.92	0.041
24. BD (1) C 10 - H 25	/160. BD*(1) C 8 - C 9		4.63	1.08	0.063
24. BD (1) C 10 - H 25	/163. BD*(1) C 9 - C 10		0.58	1.04	0.022
24. BD (1) C 10 - H 25	/164. BD*(1) C 10 - N 11		0.61	1.09	0.023
24. BD (1) C 10 - H 25	/167. BD*(1) N 11 - C 12		5.90	0.92	0.066
25. BD (1) N 11 - C 12	/ 98. RY*(1) C 10		3.00	1.50	0.060
25. BD (1) N 11 - C 12	/ 99. RY*(2) C 10		1.37	1.92	0.046
25. BD (1) N 11 - C 12	/111. RY*(2) C 13		0.68	1.74	0.031
25. BD (1) N 11 - C 12	/164. BD*(1) C 10 - N 11		1.00	1.34	0.033
25. BD (1) N 11 - C 12	/166. BD*(1) C 10 - H 25		1.78	1.23	0.042
25. BD (1) N 11 - C 12	/168. BD*(1) C 12 - C 13		1.22	1.33	0.036
25. BD (1) N 11 - C 12	/170. BD*(1) C 12 - C 17		0.88	1.26	0.030
25. BD (1) N 11 - C 12	/172. BD*(1) C 13 - C 15		2.87	1.29	0.054
25. BD (1) N 11 - C 12	/180. BD*(1) C 17 - H 24		0.82	1.24	0.029
26. BD (1) C 12 - C 13	/102. RY*(1) N 11		1.06	2.13	0.043
26. BD (1) C 12 - C 13	/114. RY*(1) N 14		0.78	2.38	0.039
26. BD (1) C 12 - C 13	/119. RY*(2) C 15		1.02	1.61	0.036

26. BD (1) C 12 - C 13	/127. RY*(2) C 17	1.57	1.89	0.049
26. BD (1) C 12 - C 13	/164. BD*(1) C 10 - N 11	1.84	1.31	0.044
26. BD (1) C 12 - C 13	/167. BD*(1) N 11 - C 12	0.99	1.14	0.030
26. BD (1) C 12 - C 13	/170. BD*(1) C 12 - C 17	2.78	1.23	0.052
26. BD (1) C 12 - C 13	/171. BD*(1) C 13 - N 14	1.94	1.18	0.043
26. BD (1) C 12 - C 13	/172. BD*(1) C 13 - C 15	4.13	1.26	0.064
26. BD (1) C 12 - C 13	/174. BD*(1) N 14 - H 27	1.76	1.15	0.040
26. BD (1) C 12 - C 13	/175. BD*(1) C 15 - N 16	4.07	1.56	0.071
26. BD (1) C 12 - C 13	/176. BD*(2) C 15 - N 16	0.95	0.82	0.025
26. BD (1) C 12 - C 13	/178. BD*(1) C 17 - N 18	2.33	1.29	0.049
27. BD (2) C 12 - C 13	/115. RY*(2) N 14	0.75	1.37	0.031
27. BD (2) C 12 - C 13	/121. RY*(4) C 15	0.51	1.05	0.022
27. BD (2) C 12 - C 13	/129. RY*(4) C 17	0.52	1.04	0.022
27. BD (2) C 12 - C 13	/165. BD*(2) C 10 - N 11	14.51	0.29	0.058
27. BD (2) C 12 - C 13	/169. BD*(2) C 12 - C 13	1.00	0.28	0.016
27. BD (2) C 12 - C 13	/177. BD*(3) C 15 - N 16	17.00	0.35	0.071
27. BD (2) C 12 - C 13	/179. BD*(2) C 17 - N 18	25.48	0.28	0.075
28. BD (1) C 12 - C 17	/103. RY*(2) N 11	0.59	2.41	0.034
28. BD (1) C 12 - C 17	/110. RY*(1) C 13	1.96	1.88	0.054
28. BD (1) C 12 - C 17	/130. RY*(1) N 18	1.23	2.11	0.046
28. BD (1) C 12 - C 17	/131. RY*(2) N 18	0.61	2.36	0.034
28. BD (1) C 12 - C 17	/167. BD*(1) N 11 - C 12	0.52	1.11	0.021
28. BD (1) C 12 - C 17	/168. BD*(1) C 12 - C 13	4.17	1.27	0.065
28. BD (1) C 12 - C 17	/171. BD*(1) C 13 - N 14	2.75	1.15	0.050
28. BD (1) C 12 - C 17	/178. BD*(1) C 17 - N 18	1.30	1.26	0.036
28. BD (1) C 12 - C 17	/180. BD*(1) C 17 - H 24	0.76	1.18	0.027
29. BD (1) C 13 - N 14	/107. RY*(2) C 12	0.79	1.93	0.035
29. BD (1) C 13 - N 14	/168. BD*(1) C 12 - C 13	2.37	1.39	0.052
29. BD (1) C 13 - N 14	/170. BD*(1) C 12 - C 17	1.79	1.32	0.044
29. BD (1) C 13 - N 14	/172. BD*(1) C 13 - C 15	1.21	1.36	0.036
29. BD (1) C 13 - N 14	/173. BD*(1) N 14 - H 26	0.55	1.25	0.023
29. BD (1) C 13 - N 14	/175. BD*(1) C 15 - N 16	2.50	1.65	0.057
29. BD (1) C 13 - N 14	/176. BD*(2) C 15 - N 16	1.70	0.91	0.035
30. BD (1) C 13 - C 15	/106. RY*(1) C 12	2.05	1.92	0.056
30. BD (1) C 13 - C 15	/114. RY*(1) N 14	0.63	2.40	0.035
30. BD (1) C 13 - C 15	/122. RY*(1) N 16	1.93	2.84	0.066
30. BD (1) C 13 - C 15	/167. BD*(1) N 11 - C 12	2.05	1.16	0.044
30. BD (1) C 13 - C 15	/168. BD*(1) C 12 - C 13	3.72	1.32	0.063

30. BD (1) C 13 - C 15	/171. BD*(1) C 13 - N 14	0.55	1.19	0.023
30. BD (1) C 13 - C 15	/173. BD*(1) N 14 - H 26	1.68	1.17	0.040
30. BD (1) C 13 - C 15	/175. BD*(1) C 15 - N 16	6.18	1.57	0.088
31. BD (1) N 14 - H 26	/111. RY*(2) C 13	2.17	1.63	0.053
31. BD (1) N 14 - H 26	/171. BD*(1) C 13 - N 14	0.79	1.09	0.026
31. BD (1) N 14 - H 26	/172. BD*(1) C 13 - C 15	3.68	1.18	0.059
31. BD (1) N 14 - H 26	/176. BD*(2) C 15 - N 16	0.63	0.74	0.019
32. BD (1) N 14 - H 27	/110. RY*(1) C 13	1.87	1.84	0.052
32. BD (1) N 14 - H 27	/168. BD*(1) C 12 - C 13	2.77	1.24	0.053
33. BD (1) C 15 - N 16	/110. RY*(1) C 13	2.37	2.24	0.065
33. BD (1) C 15 - N 16	/112. RY*(3) C 13	0.53	2.54	0.033
33. BD (1) C 15 - N 16	/118. RY*(1) C 15	2.51	1.74	0.059
33. BD (1) C 15 - N 16	/172. BD*(1) C 13 - C 15	6.52	1.59	0.091
34. BD (2) C 15 - N 16	/168. BD*(1) C 12 - C 13	3.13	0.90	0.048
34. BD (2) C 15 - N 16	/171. BD*(1) C 13 - N 14	4.83	0.77	0.055
34. BD (2) C 15 - N 16	/180. BD*(1) C 17 - H 24	0.84	0.81	0.023
35. BD (3) C 15 - N 16	/169. BD*(2) C 12 - C 13	9.77	0.35	0.057
36. BD (1) C 17 - N 18	/ 90. RY*(1) C 8	3.91	1.81	0.075
36. BD (1) C 17 - N 18	/107. RY*(2) C 12	0.60	1.98	0.031
36. BD (1) C 17 - N 18	/126. RY*(1) C 17	0.81	1.65	0.033
36. BD (1) C 17 - N 18	/158. BD*(1) N 7 - C 8	2.14	1.30	0.047
36. BD (1) C 17 - N 18	/162. BD*(1) C 8 - N 18	1.41	1.32	0.039
36. BD (1) C 17 - N 18	/168. BD*(1) C 12 - C 13	0.92	1.45	0.033
36. BD (1) C 17 - N 18	/170. BD*(1) C 12 - C 17	1.32	1.37	0.038
36. BD (1) C 17 - N 18	/180. BD*(1) C 17 - H 24	0.57	1.35	0.025
37. BD (2) C 17 - N 18	/161. BD*(2) C 8 - C 9	21.41	0.32	0.080
37. BD (2) C 17 - N 18	/169. BD*(2) C 12 - C 13	9.02	0.32	0.051
38. BD (1) C 17 - H 24	/106. RY*(1) C 12	0.67	1.69	0.030
38. BD (1) C 17 - H 24	/107. RY*(2) C 12	0.71	1.62	0.031
38. BD (1) C 17 - H 24	/130. RY*(1) N 18	0.77	1.93	0.035
38. BD (1) C 17 - H 24	/162. BD*(1) C 8 - N 18	5.61	0.96	0.066
38. BD (1) C 17 - H 24	/167. BD*(1) N 11 - C 12	6.41	0.93	0.069
38. BD (1) C 17 - H 24	/170. BD*(1) C 12 - C 17	0.53	1.02	0.021
38. BD (1) C 17 - H 24	/176. BD*(2) C 15 - N 16	0.84	0.61	0.020
38. BD (1) C 17 - H 24	/178. BD*(1) C 17 - N 18	0.57	1.08	0.022
39. CR (1) C 1	/ 66. RY*(1) C 2	0.72	11.24	0.080
39. CR (1) C 1	/ 67. RY*(2) C 2	2.21	10.81	0.138
39. CR (1) C 1	/ 69. RY*(4) C 2	0.61	11.57	0.075

39. CR (1) C 1	/ 83. RY*(2) C 6	2.71	10.85	0.153
39. CR (1) C 1	/ 85. RY*(4) C 6	0.81	11.60	0.087
39. CR (1) C 1	/134. RY*(1) H 19	1.48	10.67	0.112
39. CR (1) C 1	/144. BD*(1) C 1 - C 6	0.50	10.55	0.065
40. CR (1) C 2	/ 62. RY*(1) C 1	0.64	11.24	0.076
40. CR (1) C 2	/ 63. RY*(2) C 1	2.25	10.81	0.139
40. CR (1) C 2	/ 65. RY*(4) C 1	0.66	11.58	0.078
40. CR (1) C 2	/ 70. RY*(1) C 3	0.60	10.92	0.072
40. CR (1) C 2	/ 71. RY*(2) C 3	1.74	11.11	0.124
40. CR (1) C 2	/ 72. RY*(3) C 3	1.09	11.59	0.100
40. CR (1) C 2	/135. RY*(1) H 20	1.49	10.68	0.113
41. CR (1) C 3	/ 67. RY*(2) C 2	2.65	10.81	0.151
41. CR (1) C 3	/ 69. RY*(4) C 2	0.70	11.57	0.081
41. CR (1) C 3	/ 74. RY*(1) C 4	1.69	11.13	0.123
41. CR (1) C 3	/ 75. RY*(2) C 4	1.07	11.13	0.097
41. CR (1) C 3	/ 76. RY*(3) C 4	1.12	11.53	0.102
41. CR (1) C 3	/136. RY*(1) H 21	1.47	10.67	0.112
41. CR (1) C 3	/147. BD*(1) C 2 - C 3	0.56	10.55	0.069
41. CR (1) C 3	/150. BD*(1) C 3 - C 4	0.57	10.54	0.070
41. CR (1) C 3	/154. BD*(1) C 4 - N 7	0.88	10.37	0.086
42. CR (1) C 4	/ 70. RY*(1) C 3	3.25	10.97	0.169
42. CR (1) C 4	/ 78. RY*(1) C 5	2.92	11.26	0.162
42. CR (1) C 4	/150. BD*(1) C 3 - C 4	0.58	10.58	0.070
42. CR (1) C 4	/152. BD*(1) C 4 - C 5	1.09	10.53	0.096
42. CR (1) C 4	/158. BD*(1) N 7 - C 8	0.52	10.43	0.067
42. CR (1) C 4	/159. BD*(1) N 7 - H 23	0.69	10.40	0.076
43. CR (1) C 5	/ 74. RY*(1) C 4	2.55	11.13	0.151
43. CR (1) C 5	/ 82. RY*(1) C 6	1.30	11.22	0.108
43. CR (1) C 5	/ 83. RY*(2) C 6	1.59	10.85	0.117
43. CR (1) C 5	/ 95. RY*(2) C 9	2.04	11.32	0.136
43. CR (1) C 5	/152. BD*(1) C 4 - C 5	0.75	10.49	0.080
43. CR (1) C 5	/163. BD*(1) C 9 - C 10	0.63	10.48	0.073
44. CR (1) C 6	/ 62. RY*(1) C 1	0.52	11.24	0.068
44. CR (1) C 6	/ 63. RY*(2) C 1	2.64	10.81	0.151
44. CR (1) C 6	/ 65. RY*(4) C 1	0.68	11.57	0.079
44. CR (1) C 6	/ 78. RY*(1) C 5	0.53	11.21	0.069
44. CR (1) C 6	/ 79. RY*(2) C 5	2.10	11.36	0.138
44. CR (1) C 6	/ 80. RY*(3) C 5	0.93	11.65	0.093

44. CR (1) C 6	/137. RY*(1) H 22	1.47	10.69	0.112
44. CR (1) C 6	/144. BD*(1) C 1 - C 6	0.53	10.55	0.067
44. CR (1) C 6	/155. BD*(1) C 5 - C 6	0.54	10.53	0.068
44. CR (1) C 6	/156. BD*(1) C 5 - C 9	0.87	10.48	0.086
45. CR (1) N 7	/ 75. RY*(2) C 4	3.88	15.27	0.217
45. CR (1) N 7	/ 76. RY*(3) C 4	0.67	15.67	0.092
45. CR (1) N 7	/ 86. RY*(1) N 7	0.69	16.05	0.094
45. CR (1) N 7	/ 90. RY*(1) C 8	1.44	15.03	0.132
45. CR (1) N 7	/ 91. RY*(2) C 8	2.82	15.15	0.185
45. CR (1) N 7	/ 92. RY*(3) C 8	0.57	15.63	0.084
45. CR (1) N 7	/138. RY*(1) H 23	0.78	14.90	0.096
45. CR (1) N 7	/158. BD*(1) N 7 - C 8	0.52	14.52	0.078
46. CR (1) C 8	/ 90. RY*(1) C 8	0.95	10.99	0.091
46. CR (1) C 8	/ 94. RY*(1) C 9	3.04	11.37	0.166
46. CR (1) C 8	/ 96. RY*(3) C 9	0.60	11.72	0.075
46. CR (1) C 8	/154. BD*(1) C 4 - N 7	0.61	10.46	0.072
46. CR (1) C 8	/159. BD*(1) N 7 - H 23	0.68	10.45	0.075
46. CR (1) C 8	/160. BD*(1) C 8 - C 9	1.28	10.62	0.105
46. CR (1) C 8	/163. BD*(1) C 9 - C 10	0.50	10.58	0.066
46. CR (1) C 8	/178. BD*(1) C 17 - N 18	0.92	10.61	0.088
47. CR (1) C 9	/ 78. RY*(1) C 5	0.52	11.21	0.068
47. CR (1) C 9	/ 79. RY*(2) C 5	1.53	11.36	0.118
47. CR (1) C 9	/ 80. RY*(3) C 5	0.70	11.65	0.081
47. CR (1) C 9	/ 90. RY*(1) C 8	2.97	10.89	0.161
47. CR (1) C 9	/ 92. RY*(3) C 8	0.94	11.49	0.093
47. CR (1) C 9	/ 98. RY*(1) C 10	2.05	10.69	0.132
47. CR (1) C 9	/101. RY*(4) C 10	0.65	11.43	0.077
47. CR (1) C 9	/155. BD*(1) C 5 - C 6	0.81	10.53	0.083
47. CR (1) C 9	/160. BD*(1) C 8 - C 9	0.73	10.51	0.079
47. CR (1) C 9	/162. BD*(1) C 8 - N 18	0.86	10.39	0.085
48. CR (1) C 10	/ 94. RY*(1) C 9	1.01	11.33	0.096
48. CR (1) C 10	/ 95. RY*(2) C 9	0.90	11.38	0.090
48. CR (1) C 10	/ 96. RY*(3) C 9	1.12	11.69	0.102
48. CR (1) C 10	/103. RY*(2) N 11	0.64	11.73	0.077
48. CR (1) C 10	/140. RY*(1) H 25	1.56	10.78	0.116
48. CR (1) C 10	/167. BD*(1) N 11 - C 12	1.39	10.42	0.108
49. CR (1) N 11	/ 98. RY*(1) C 10	4.74	14.76	0.237
49. CR (1) N 11	/ 99. RY*(2) C 10	0.90	15.17	0.105

49. CR (1) N 11	/101. RY*(4) C 10	0.66	15.50	0.090
49. CR (1) N 11	/102. RY*(1) N 11	0.75	15.42	0.096
49. CR (1) N 11	/106. RY*(1) C 12	0.96	15.19	0.108
49. CR (1) N 11	/107. RY*(2) C 12	2.83	15.12	0.185
50. CR (1) C 12	/111. RY*(2) C 13	2.72	10.98	0.154
50. CR (1) C 12	/112. RY*(3) C 13	1.09	11.49	0.100
50. CR (1) C 12	/126. RY*(1) C 17	2.46	10.78	0.146
50. CR (1) C 12	/128. RY*(3) C 17	0.74	11.51	0.082
50. CR (1) C 12	/164. BD*(1) C 10 - N 11	0.80	10.58	0.082
50. CR (1) C 12	/168. BD*(1) C 12 - C 13	1.18	10.57	0.100
50. CR (1) C 12	/172. BD*(1) C 13 - C 15	0.57	10.53	0.070
51. CR (1) C 13	/106. RY*(1) C 12	1.49	11.21	0.116
51. CR (1) C 13	/107. RY*(2) C 12	1.81	11.14	0.127
51. CR (1) C 13	/108. RY*(3) C 12	0.89	11.65	0.091
51. CR (1) C 13	/116. RY*(3) N 14	0.63	11.33	0.075
51. CR (1) C 13	/118. RY*(1) C 15	1.93	10.72	0.129
51. CR (1) C 13	/120. RY*(3) C 15	1.16	11.55	0.104
51. CR (1) C 13	/168. BD*(1) C 12 - C 13	1.15	10.61	0.099
51. CR (1) C 13	/174. BD*(1) N 14 - H 27	0.63	10.46	0.072
51. CR (1) C 13	/175. BD*(1) C 15 - N 16	2.29	10.86	0.141
52. CR (1) N 14	/110. RY*(1) C 13	1.26	15.25	0.124
52. CR (1) N 14	/111. RY*(2) C 13	3.49	15.05	0.205
52. CR (1) N 14	/112. RY*(3) C 13	1.14	15.56	0.119
52. CR (1) N 14	/116. RY*(3) N 14	0.53	15.37	0.081
52. CR (1) N 14	/141. RY*(1) H 26	1.08	14.95	0.114
52. CR (1) N 14	/142. RY*(1) H 27	0.85	14.85	0.100
52. CR (1) N 14	/171. BD*(1) C 13 - N 14	0.54	14.52	0.080
53. CR (1) C 15	/110. RY*(1) C 13	3.35	11.20	0.173
53. CR (1) C 15	/118. RY*(1) C 15	1.73	10.70	0.122
53. CR (1) C 15	/124. RY*(3) N 16	0.66	11.94	0.079
53. CR (1) C 15	/175. BD*(1) C 15 - N 16	0.51	10.84	0.066
54. CR (1) N 16	/118. RY*(1) C 15	12.93	14.64	0.390
54. CR (1) N 16	/172. BD*(1) C 13 - C 15	2.89	14.49	0.184
55. CR (1) C 17	/106. RY*(1) C 12	2.27	11.19	0.143
55. CR (1) C 17	/108. RY*(3) C 12	0.89	11.63	0.091
55. CR (1) C 17	/126. RY*(1) C 17	0.53	10.80	0.067
55. CR (1) C 17	/131. RY*(2) N 18	0.70	11.69	0.081
55. CR (1) C 17	/139. RY*(1) H 24	1.66	10.83	0.120

55. CR (1) C 17	/162. BD*(1) C 8 - N 18	1.37	10.47	0.107
55. CR (1) C 17	/167. BD*(1) N 11 - C 12	0.58	10.43	0.070
56. CR (1) N 18	/ 90. RY*(1) C 8	2.31	14.95	0.166
56. CR (1) N 18	/ 91. RY*(2) C 8	2.64	15.07	0.178
56. CR (1) N 18	/126. RY*(1) C 17	4.13	14.79	0.221
56. CR (1) N 18	/127. RY*(2) C 17	1.05	15.17	0.113
56. CR (1) N 18	/128. RY*(3) C 17	0.64	15.52	0.089
56. CR (1) N 18	/130. RY*(1) N 18	0.95	15.42	0.108
56. CR (1) N 18	/160. BD*(1) C 8 - C 9	0.61	14.58	0.085
57. LP (1) N 7	/153. BD*(2) C 4 - C 5	35.12	0.28	0.090
57. LP (1) N 7	/161. BD*(2) C 8 - C 9	46.56	0.27	0.101
58. LP (1) N 11	/ 98. RY*(1) C 10	4.30	1.04	0.061
58. LP (1) N 11	/ 99. RY*(2) C 10	0.53	1.46	0.025
58. LP (1) N 11	/106. RY*(1) C 12	0.84	1.47	0.032
58. LP (1) N 11	/107. RY*(2) C 12	1.43	1.41	0.041
58. LP (1) N 11	/163. BD*(1) C 9 - C 10	11.06	0.83	0.087
58. LP (1) N 11	/166. BD*(1) C 10 - H 25	4.44	0.77	0.053
58. LP (1) N 11	/168. BD*(1) C 12 - C 13	2.68	0.87	0.044
58. LP (1) N 11	/170. BD*(1) C 12 - C 17	9.68	0.80	0.080
58. LP (1) N 11	/173. BD*(1) N 14 - H 26	5.39	0.72	0.057
59. LP (1) N 14	/169. BD*(2) C 12 - C 13	51.42	0.25	0.105
60. LP (1) N 16	/118. RY*(1) C 15	18.95	1.18	0.134
60. LP (1) N 16	/172. BD*(1) C 13 - C 15	9.58	1.03	0.089
61. LP (1) N 18	/ 90. RY*(1) C 8	2.61	1.24	0.052
61. LP (1) N 18	/ 91. RY*(2) C 8	1.36	1.36	0.039
61. LP (1) N 18	/126. RY*(1) C 17	4.00	1.08	0.060
61. LP (1) N 18	/127. RY*(2) C 17	0.71	1.47	0.030
61. LP (1) N 18	/158. BD*(1) N 7 - C 8	6.32	0.73	0.062
61. LP (1) N 18	/160. BD*(1) C 8 - C 9	10.24	0.87	0.086
61. LP (1) N 18	/170. BD*(1) C 12 - C 17	11.46	0.80	0.087
61. LP (1) N 18	/180. BD*(1) C 17 - H 24	3.99	0.78	0.051
145. BD*(2) C 1 - C 6	/ 64. RY*(3) C 1	1.05	0.77	0.064
145. BD*(2) C 1 - C 6	/ 84. RY*(3) C 6	1.10	0.77	0.066
148. BD*(2) C 2 - C 3	/ 68. RY*(3) C 2	1.15	0.78	0.065
148. BD*(2) C 2 - C 3	/ 73. RY*(4) C 3	1.19	0.77	0.066
153. BD*(2) C 4 - C 5	/ 77. RY*(4) C 4	0.96	0.81	0.050
153. BD*(2) C 4 - C 5	/ 81. RY*(4) C 5	1.23	0.85	0.058
153. BD*(2) C 4 - C 5	/145. BD*(2) C 1 - C 6	194.02	0.02	0.079

153. BD*(2) C 4 - C 5	/148. BD*(2) C 2 - C 3	308.27	0.01	0.080
161. BD*(2) C 8 - C 9	/ 93. RY*(4) C 8	0.75	0.78	0.046
161. BD*(2) C 8 - C 9	/ 97. RY*(4) C 9	1.03	0.87	0.057
169. BD*(2) C 12 - C 13	/109. RY*(4) C 12	0.91	0.83	0.054
169. BD*(2) C 12 - C 13	/113. RY*(4) C 13	0.81	0.82	0.050
169. BD*(2) C 12 - C 13	/177. BD*(3) C 15 - N 16	36.61	0.07	0.088
179. BD*(2) C 17 - N 18	/129. RY*(4) C 17	0.54	0.76	0.052

a.u: Atomic units.

[a]: Energy of hyper conjugative interaction (Stabilization energy).

[b]: Energy difference between donor (i) and acceptor (j) NBO orbitals.

[c]: F(i,j) is the Fock matrix element between i and j NBO orbitals.

8. References:

1 R. G. Parr and W. Yang, *Density Functional Theory of Atoms and Molecules*, Oxford University Press, Oxford, 1989.

2 (*a*) V. Barone and M. Cossi ,*J. Phys. Chem. A,* 1998, **102**, 1995; (*b*) M. Cossi and V. Barone, *J. Chem. Phys.,* 2001, **115**, 4708; (*c*) M. Cossi , N. Rega, G. Scalmani and V. Barone*J. Comp. Chem.,* 2003, **24**, 669.

3 A. D. Becke, *J. Chem. Phys.,* 1993, **98**, 5648.

4 C. Lee , W. Yang and R. G. Parr, *Phys. Rev. B*, 1998, **37**, 785.

5 M. E. Casida, C. Jamoroski , K. C. Casida and D. R. Salahub,*J. Chem. Phys.,* 1998, **108**, 4439; R. E. Stratmann, G. E. Scuseria, M. J. Frisch, *J. Chem. Phys.,* 1998, **109**, 8218; R. Bauernschmitt and R. Ahlrichs, *Chem. Phys. Lett.,* 1996, **256**, 454.

6 M. J. Frisch, G. W. Trucks, H. B. Schlegel, G. E. Scuseria, M. A. Robb, J. R. Cheeseman, G. Scalmani, V. Barone, B. Mennucci, G. A. Petersson, H. Nakatsuji, M. Caricato, X. Li, H. P. Hratchian, A. F. Izmaylov, J. Bloino, G. Zheng, J. L. Sonnenberg, M. Hada, M. Ehara, K. Toyota, R. Fukuda, J. Hasegawa, M. Ishida, T. Nakajima, Y. Honda, O. Kitao, H. Nakai, T. Vreven, J. A. Montgomery Jr., J. E. Peralta, F. Ogliaro, M. Bearpark, J. J. Heyd, E. Brothers, K. N. Kudin, V. N. Staroverov, R. Kobayashi, J. Normand, K. Raghavachari, A. Rendell, J. C. Burant, S. S. Iyengar, J. Tomasi, M. Cossi, N. Rega, J. M. Millam, M. Klene, J. E. Knox, J. B. Cross, V. Bakken, C. Adamo, J. Jaramillo, R. Gomperts, R. E. Stratmann, O. Yazyev, A. J. Austin, R. Cammi, C. Pomelli, J. W. Ochterski, R. L. Martin, K. Morokuma, V. G. Zakrzewski, G. A. Voth, P. Salvador, J. J. Dannenberg, S. Dapprich, A. D. Daniels, Ö. Farkas, J. B. Foresman, J. V Ortiz, J. Cioslowski and D. J. Fox, Gaussian Inc., 2009, Wallingford CT.

7 N. M. O'Boyle, A. L. Tenderholt and K. M. Langner, *J. Comp. Chem.,* 2008, **29**, 839.
